# Epidemiological surveillance reveals major pathogen dynamics in Brazilian Nile tilapia aquaculture from 2023 to 2025

**DOI:** 10.3389/fvets.2026.1944605

**Published:** 2026-10-19

**Authors:** Mateus Cardoso-Guimarães, Miguel Frederico Fernandez-Alarcon, Rodolfo Correa, Mariene Miyoko-Natori, Renata Galetti, Adrielli Brito de Souza, Ana Cristina Squizatto, Rafael Silva, Luana Araújo de Almeida, Thaise Mota Sátiro, Ricardo Ildefonso, Marco Rozas-Serri

**Affiliations:** Pathovet Labs, Ribeirão Preto, Brazil

**Keywords:** antimicrobial stewardship, aquaculture health, disease monitoring, multi-pathogen dynamics, *Oreochromis niloticus*, time-series forecasting

## Abstract

**Introduction:**

Brazil is among the world’s largest producers of Nile tilapia (*Oreochromis niloticus*), a species increasingly challenged by a complex of cocirculating pathogens that threatens production sustainability.

**Methods:**

We conducted a structured epidemiological surveillance program across the three main tilapia-producing macro-regions of Brazil (Southeast, Central-West, and Northeast) between 2023 and 2025. A total of 26,057 diagnostic samples, generating 68,514 pathogen-specific tests, were analyzed for *Aeromonas* spp., *Edwardsiella* spp., *Flavobacterium* spp., *Francisella orientalis*, Infectious Spleen and Kidney Necrosis Virus (ISKNV), *Lactococcus* spp., and *Streptococcus* spp. using standardized bacteriological, real-time PCR, and MALDI-TOF MS protocols, and antimicrobial susceptibility (minimum inhibitory concentration, MIC) was determined for four antimicrobials. Generalized linear models (with a farm-clustered mixed-model sensitivity analysis), time-series analysis (ADF stationarity, ARIMA), co-detection network analysis, and Gamma-log MIC modelling were applied.

**Results:**

The cumulative overall detection positivity rate (DPR) was 18.1%, with *Flavobacterium* spp. showing the highest cumulative positivity (21.6%) on the smallest testing base, and *Lactococcus* spp. (18.4%), ISKNV (23.3%), and *Streptococcus* spp. (15.2%) representing the dominant, most widely sampled agents; *S. agalactiae* serotype Ia was not detected by serotype-specific qPCR. Detection was structured principally by macro-region and pathogen-specific temporal dynamics, whereas host developmental stage exerted a heterogeneous, generally weak influence. Five of the seven pathogens showed stationary, endemically compatible dynamics; *Streptococcus* spp. was non-stationary, consistent with active geographic expansion into the Northeast, whereas non-stationarity in *Flavobacterium* spp. reflected sampling instability rather than directional change. Quarterly aggregation minimized forecast error among the frequencies evaluated. Co-detection was predominantly independent, with only a weak module linking the three dominant agents. Antimicrobial resistance was structured by pathogen and antibiotic, with significant pathogen-specific interactions, and the fry stage showed higher MICs.

**Discussion:**

These findings reframe the historically *Streptococcus*-centered view of Brazilian tilapia health and, within the constraints of a structured but non-probabilistic design, provide a descriptive, data-driven baseline to inform regionalized early-warning systems and rational antimicrobial stewardship in Brazilian tilapia aquaculture.

## 1 Introduction

Nile tilapia (*Oreochromis niloticus*) is one of the most economically important farmed fish species in the world, with global production reaching approximately 5.3 million tons annually, ranking it among the top five aquaculture species worldwide (1). Brazil has emerged as the fourth-largest tilapia producer globally, with the species accounting for 55–57% of the country’s total aquaculture output (2).

Over the past decade, the Brazilian tilapia industry has experienced a compound annual growth rate exceeding 10%, driven by expansion of cage culture systems in large reservoirs and intensification of production across multiple macro-regions (2). However, this growth trajectory has been accompanied by a parallel escalation in the diversity and complexity of infectious diseases affecting farmed tilapia populations, posing a substantial biosecurity challenge for producers and veterinary professionals alike (3).

Streptococcosis, caused by *Streptococcus agalactiae* (Lancefield’s group B streptococcus, GBS), is one of the most important and economically damaging bacterial diseases in Brazilian tilapia farming, where it has been recognized as a major pathogen since its first reports in the mid-2000s (4). Outbreaks are concentrated in the warm summer months, when water temperature exceeds 27 °C, and are favored by intensive culture in floating cages, high stocking densities, and the stress associated with poor water quality. In Brazil, serotype Ib has historically been the predominant one, although strains of serotypes Ia and III have also been described, including the emergence of highly virulent, multidrug-resistant lineages (5).

*Lactococcus petauri* (and to a lesser extent *L. garvieae*) was first associated with mass mortality outbreaks in Brazilian cage-farmed tilapia starting in 2020, with lactococcosis dispersing rapidly across different river basins and causing cumulative losses of 15–35% in affected grow-out units (6). The predominant circulating lineage in Brazil was identified as *L. petauri* sequence type ST24, with pathogenicity challenges confirming an estimated LD₅₀ of 5.74 × 10^3^ CFU/fish in juvenile Nile tilapia.

*Aeromonas hydrophila* and related species have long been recognized as opportunistic pathogens associated with motile Aeromonas septicemia (MAS) in stressed fish populations (7). *Aeromonas veronii* has been reported as an emerging bacterial pathogen in cage-farmed Nile tilapia in Brazil, from a high-mortality outbreak in late 2022 (3). Isolates identified by *16S rRNA*, *gyrB*, and *rpoB* sequencing showed a multidrug-resistant phenotype, susceptible only to florfenicol and resistant to oxytetracycline, enrofloxacin, and amoxicillin, with the resistance genes *qnrS2* and *tetA* confirmed genotypically. The strain was highly pathogenic to juvenile tilapia, reaching 100% mortality at the highest doses, illustrating how sporadic emergence of multidrug-resistant pathogens can compound Brazil’s established tilapia disease complex.

*Flavobacterium columnare* (and related species recently reclassified into *F. davisii*, *F. covae*, and *F. oreochromis*) causes columnaris disease, one of the leading causes of bacterial mortality in tilapia and other freshwater fish in Brazil (8). *Edwardsiella* spp. represent an additional concern, with *E. tarda* and the recently described *E. anguillarum* showing increasing antimicrobial resistance profiles that complicate treatment options (9).

Concurrently, Infectious Spleen and Kidney Necrosis Virus (ISKNV, Clade 1), a megalocytivirus of the family Iridoviridae, was first reported causing fatal outbreaks in Brazilian tilapia farms in June 2020 (10). ISKNV has been identified not only in co-infections with *S. agalactiae*, *E. tarda*, and *A. hydrophila* (10), but also alongside multidrug-resistant *A. veronii* in farmed tilapia (3), illustrating the growing diversity of pathogens circulating together in this production system.

Despite the well-documented emergence of these pathogens, structured longitudinal epidemiological data across macro-regions and production stages remain critically lacking for Brazilian tilapia, with most studies limited to outbreak investigations or single-farm analyses. This gap leaves stakeholders without baselines to distinguish endemic fluctuation from emerging-disease signals, calibrate sampling, or apply antimicrobials rationally. Concerns over antimicrobial resistance (AMR) are amplified because only florfenicol and oxytetracycline are authorized for tilapia in Brazil (11), yet enrofloxacin and amoxicillin residues in commercial fillets indicate off-label use that may accelerate resistance (12).

Antimicrobial resistance (AMR) adds a further, cross-cutting dimension to this disease complex. Intensive tilapia systems combine high stocking densities, recurrent bacterial challenge, and, in many settings, limited access to licensed veterinary antimicrobials, which together create strong selective pressure for resistant bacterial populations. Reports from tilapia and other warm-water aquaculture systems document rising resistance in *Aeromonas*, *Streptococcus*, *Edwardsiella*, and *Lactococcus* to the few compounds available, and the recovery of multidrug-resistant strains carrying transferable resistance determinants 3,9,12. Because aquaculture shares water, feed, and trade networks with terrestrial food systems, AMR in farmed fish is increasingly framed as a One Health concern, in which resistant bacteria and their resistance genes may disseminate to the environment and along the food chain. Quantifying pathogen-specific susceptibility, and the conditions under which resistance emerges, is therefore integral to any modern surveillance framework for tilapia, yet such data remain scarce and fragmentary for the Brazilian sector.

A recent study provided a spatiotemporal assessment of *S. agalactiae* and *Francisella orientalis* in Brazilian tilapia, confirming the endemic, temperature-driven, and serotype-structured nature of these pathogens (13). However, that survey relied on outbreak-driven, autogenous vaccine-oriented bacteriological sampling restricted to two bacterial agents, with uneven regional coverage and without viral or antimicrobial-resistance data, leaving the broader co-circulating pathogen complex of Brazilian tilapia largely uncharacterized at the population level. A comprehensive, multi-pathogen surveillance baseline that integrates bacterial, viral, and antimicrobial-resistance data across production stages is therefore still lacking.

In response to these gaps, the present study describes the outcomes of a multi-year epidemiological surveillance program covering the three main tilapia-producing macro-regions of Brazil (Southeast, Central-West, and Northeast) between 2023 and 2025. The specific objectives were: (1) to estimate pathogen-specific DPR stratified by region, year, and production stage; (2) to identify the principal epidemiological determinants of DPR variation through generalized linear modeling; (3) to characterize the temporal dynamics of each pathogen using time-series analysis with the aim of defining optimal surveillance intervals; and (4) to quantify antimicrobial susceptibility profiles (MIC) for the most clinically relevant pathogen-antimicrobial combinations.

## 2 Materials and methods

### 2.1 Ethical and animal welfare compliance

This was a retrospective study based on diagnostic records generated during routine commercial health-monitoring of Nile tilapia farms in Brazil between 2023 and 2025. No animals were sampled specifically for this study; all clinical sampling had been performed by the attending veterinary services as part of standard diagnostic practice. The CEUA-Pathovet Labs Ethics Committee reviewed the study and determined that, as a retrospective analysis of pre-existing diagnostic records from field studies conducted under commercial production conditions, it did not require formal committee approval. This determination was issued by the committee prior to submission of the manuscript (CEUA-Pathovet Labs, Protocol No. 05/2026, March 2026). This position is consistent with recognized criteria for field research on fishes, under which collection and diagnostic studies conducted under commercial conditions frequently fall outside the scope of formal Animal Ethics Committee review (14).

### 2.2 Study design and sampling

A prospective longitudinal surveillance program was conducted from January 2023 to December 2025 across the three major tilapia-producing macro-regions of Brazil: Southeast (SE), Central-West (CW), and Northeast (NE). To increase geographic representativeness and stabilize variance, the five legal administrative regions of Brazil (North, Northeast, Central-West, Southeast, and South) were consolidated into these three study macro-regions: the Northern state sampled (Tocantins) was grouped with the Northeast, and the Southern states (Paraná and Santa Catarina) with the Southeast. Pathogen detection rates are reported as detection positivity rate (DPR), or apparent prevalence, derived from a structured but non-probabilistic surveillance scheme. Because production units were enrolled through voluntary participation, the reported values may not reflect true infection prevalence at the national or regional population level, and no correction for diagnostic sensitivity and specificity was applied. Inter-regional and inter-annual comparisons should therefore be interpreted as relative trends rather than as absolute, population-level prevalence estimates.

DPR values are reported at the genus level (*Aeromonas* spp., *Edwardsiella* spp., *Flavobacterium* spp., *Lactococcus* spp., and *Streptococcus* spp.) rather than by species or serotype. This aggregation follows from the structure of the diagnostic protocols applied: detection relied on a combination of species-specific qPCR assays and MALDI-TOF MS identification (Sections 2.3.1–2.3.2), whose resolution varied among agents and among sampling occasions according to the diagnostic request submitted. Genus-level aggregation harmonizes records generated through heterogeneous identification pathways into a consistent unit of observation, maximizing the number of records available for statistical modelling and enabling comparable time-series and co-detection analyses across all seven pathogen agents. Where species- or serotype-level information was available it is reported descriptively (Section 3.1) but was not used as the modelling unit. This approach prioritizes analytical coverage and inter-pathogen comparability over taxonomic granularity. DPR values should accordingly be read as genus-level indicators of pathogen circulation rather than as species-specific prevalence estimates.

Samples were collected during routine health monitoring visits to commercial tilapia production units, comprising both net-pen cage and earthen pond systems. Fish showing clinical signs of disease, lethargy, exophthalmia, skin ulceration, abnormal swimming behavior, or hemorrhage, were sampled together with a random selection of apparently healthy individuals, so that DPR would reflect the broader infection status of each production unit rather than being restricted to clinically affected fish. Each fish was assigned at the time of collection to one of three production stages: fry (0.5–10 g), juvenile (10–300 g), or adult (> 300 g).

The total sampling effort comprised 26,057 individual diagnostic records, distributed as 6,639 in 2023, 9,163 in 2024, and 10,255 in 2025, reflecting a progressive expansion of the surveillance network over the study period. The SE macro-region accounted for the largest share, with 19,696 samples (75.6% of the total), followed by the NE (*n* = 3,460) and CW (*n* = 2,901). Sampling, clinical examination, euthanasia, and transport procedures strictly adhered to institutional standard operating protocols for field disease investigation and diagnostic sampling, as outlined in the guidelines of the American Fisheries Society Committee on the Use of Fish in Research (15). Fish were euthanized by overdose of tricaine methanesulfonate (MS-222) in accordance with the American Veterinary Medical Association (AVMA) Guidelines for the Euthanasia of Animals and institutional animal care protocols.

The surveillance network comprised commercial Nile tilapia production units distributed across 16 Brazilian states, consolidated into the three study macro-regions as described above. In total, the network included 280 distinct companies (empresas) operating at 154 distinct culture sites, located in the main tilapia-producing states and their associated reservoir and river-basin systems. By study macro-region, the Southeast (São Paulo, Minas Gerais, Espírito Santo, Paraná, and Santa Catarina) contributed the largest share (220 companies, 112 sites), followed by the Central-West (Mato Grosso do Sul, Goiás, Distrito Federal, Mato Grosso; 35 companies, 19 sites) and the Northeast (Bahia, Pernambuco, Alagoas, Ceará, Piauí, Maranhão, and Tocantins; 25 companies, 23 sites); the full state-level breakdown is provided in Supplementary Table S1. Because several companies operated within shared culture-site localities (e.g., major reservoirs or river stretches), the number of companies exceeded the number of culture sites in some states; the culture site is the unit represented by the Site random effect in the hierarchical models. Units were sampled during routine health-monitoring submissions across the three-year period, and a substantial subset of farms was sampled repeatedly, so that the dataset combines cross-sectional and repeated-measures records. This repeated within-farm sampling is the reason a farm/site random effect was included in the hierarchical sensitivity analysis (Section 2.4.2; Supplementary Table S1).

### 2.3 Pathogen detection methods

#### 2.3.1 Bacteriological isolation and identification

For microbiological analyses, sampling was targeted, collecting only fish that showed clinical signs consistent with infectious diseases. The number of fish sampled was between 3 and 5 individuals per tank or net pen, without a fixed sample size. The basic bacteriological procedure consisted of aseptically sampling different organs of each fish (spleen, kidneys, liver) with a sterile loop and streaking a standard 10 μL loopful of tissue exudate from each organ onto Brain Heart Infusion (BHI) agar supplemented with 5% defibrinated sheep blood (Heel do Brazil Biomedica Ltda., São José dos Pinhais, PR, Brazil) incubated at 28 °C for 24–72 h, as a broad and routine diagnostic medium for the primary isolation of *Aeromonas, Streptococcus*, *Lactococcus*, and *Edwardsiella*. BHI medium is a non-selective enriched medium that supports the growth of these common fish pathogens, facilitating their primary isolation from infected tissues. At the same time, the homogenate was streaked onto cysteine heart agar supplemented with 2% bovine hemoglobin (CHAB) for *F. orientalis* (16). Plates were incubated at 22–28 °C in ambient atmosphere for 48–72 h. Colonies typically appeared as small, grayish, smooth, Gram-negative coccobacilli on CHAB.

Colonies exhibiting characteristic morphology were subcultured for purification prior to identification. Species-level identification was confirmed using Matrix-Assisted Laser Desorption/Ionization Time-of-Flight Mass Spectrometry (MALDI-TOF MS), an established rapid and cost-effective method that generates protein spectra from bacterial colonies for comparison against reference databases (17). Briefly, a fresh colony from each isolate was spotted onto a steel target plate, overlaid with 1 μL of 70% formic acid and 1 μL of α-cyano-4-hydroxycinnamic acid matrix solution, and air-dried. Mass spectra were acquired using a Bruker Microflex MALDI-TOF Biotyper system (Bruker Corporation, Billerica, MA, USA) and compared against a reference database for fish bacterial pathogens to obtain species-level identifications by using MALDI Biotyper RTC software (Bruker®). Species-level identification is accepted when log-score values meet manufacturer criteria (≥2.0) (18).

#### 2.3.2 Real-time PCR identification from tissue samples

For molecular analyses, two request types were distinguished. Targeted molecular testing was performed on fish showing clinical signs, as part of active outbreak or disease investigations; random molecular testing was performed on apparently healthy fish submitted for routine health-status monitoring or certification. Both request types followed the same laboratory workflow, and each molecular record was labelled by request type so that the two could be distinguished analytically. Samples of approximately 0.3 to 0.5 cm^3^ in volume were aseptically collected from the heart, liver, kidneys, and spleen of each fish. Following collection, specimens were immediately immersed in 70% ethanol maintained at a 1:10 sample-to-fixative ratio and preserved for 48–72 h at room temperature to ensure adequate fixation prior to laboratory processing.

Then, tissue samples were arranged into pools of 3–5 fish for DNA extraction. All fish within a given pool originated from the same farm, cage or tank, sampling date, and production stage, so that each molecular pool constituted a single, internally homogeneous diagnostic record; pools were never assembled across farms, dates, or developmental stages. Genomic DNA isolation was performed through a two-stage process. Initially, tissue samples were mechanically disrupted using magnetic bead-based homogenization with the L-Beader 24 tissue disruptor (Loccus do Brasil Ltda, Cotia, SP, Brazil) according to manufacturer specifications. Subsequently, nucleic acid extraction was accomplished semi-automatically using the Maxwell® RSC instrument (Promega Corporation, Madison, WI, USA) in conjunction with the RSC Genomic DNA Kit, following the manufacturer’s established protocol. Prior to automated extraction, each tissue-pool homogenate was pre-digested by combining it with 1 mL of phosphate-buffered saline (pH 7.4), 200 μL of lysis buffer, and 20 μL of proteinase K, and incubating until lysis was complete; the resulting lysate (100 μL per pool) was then carried into the Maxwell® RSC Genomic DNA extraction described above. Proteinase K and lysis buffer were thus added during sample preparation, not to the purified DNA.

The presence of enzootic pathogens included in the monitoring plan was routinely assessed by quantitative PCR for ISKNV (19), *S. agalactiae* using serotype-specific qPCR assays that discriminate serotypes Ia, Ib, and III (20–22), *S. iniae* (20), *L. garvieae*/*petauri* (23), *A. hydrophila* (24), *F. orientalis* (25), *E. tarda* and *E. anguillarum* (26), and *F. columnare* (27). Serotype assignment for *S. agalactiae* therefore relied on validated serotype-specific qPCR. Quantitative real-time PCR (qPCR) reactions were assembled in a final volume of 10 μL per sample, comprising 5 μL of 2X GoTaq qPCR Master Mix (Promega Corporation, Madison, WI, USA), 1.4 μL of each primer at a final concentration of 300 nM, and 1.5 μL of template DNA in duplicate replicates. Amplification was performed using the QuantStudio 3 Real-Time PCR System (Applied Biosystems, Life Technologies, Carlsbad, CA, USA) with the following thermal cycling parameters: initial denaturation at 95 °C for 2 min, followed by 40 cycles of denaturation at 95 °C for 15 s and annealing/extension at 60 °C for 60 s.

Quality assurance was maintained through the inclusion of positive controls (genomic DNA), negative controls consisting of ultrapure water (UPW) without template DNA, and negative extraction blanks in every amplification run. Endogenous extraction efficiency was monitored through concurrent amplification of the Nile tilapia β-actin reference gene (28). Cycling threshold (Ct) values were manually determined and recorded with a maximum detection ceiling of 40 Ct, with threshold consistency verified across all experimental runs. Samples were classified as positive at Ct values below 35, as indeterminate at Ct values between 35 and 40, and as negative in samples yielding no detectable Ct signal (No-Ct), consistent with established analytical criteria for qPCR-based diagnostics in aquatic animal pathogens (29).

#### 2.3.3 Antimicrobial susceptibility testing

Antimicrobial susceptibility profiling was performed by disk diffusion following the general procedures of the Clinical and Laboratory Standards Institute, applying the aquatic-animal-specific documents VET03 (disk diffusion) and VET04 (broth dilution and interpretation, including quality control) where applicable, and the human M100 tables (30) only as a supplementary reference for antimicrobial–organism pairs without an aquatic-animal breakpoint. Bacterial cultures grown overnight were standardized by suspension in 0.85% sterile saline and adjusted to a 0.5 McFarland optical density. Mueller–Hinton agar (MHA) plates were inoculated using sterile cotton applicators to ensure uniform bacterial distribution. Commercially prepared antimicrobial disks matching the agents evaluated in the minimum inhibitory concentration (MIC) assay were aseptically positioned on the inoculated agar: oxytetracycline (OTC, 30 μg), florfenicol (FFC, 30 μg), amoxicillin (AMX, 10 μg), and enrofloxacin (ENR, 5 μg). Plates were incubated at 28 ± 2 °C for 18–24 h under aerobic conditions, and zones of inhibition were measured in millimetres (31). Because CLSI aquatic-animal interpretive criteria are organism-specific and are defined for a limited set of pathogen–drug combinations, categorical susceptibility was assigned only where a validated aquatic-animal breakpoint or epidemiological cut-off value existed for that specific pathogen–antimicrobial pair; where none was available, MIC and zone values were reported numerically without categorical classification, and no claim of resistance was made from MIC magnitude alone.

#### 2.3.4 Minimum inhibitory concentration determination

Quantitative antimicrobial susceptibility assessment of isolates from fish was conducted utilizing the broth microdilution reference methodology as prescribed by CLSI guidelines with species-appropriate modifications. Determinations were performed in accordance with CLSI M07 protocols (32). Cation-adjusted Mueller–Hinton broth (CAMHB) served as the culture medium for all susceptibility assays. The four antimicrobial agents under evaluation (OTC, FFC, ENR, AMX) were prepared as concentrated stock solutions immediately prior to panel assembly, with serial dilutions accomplished in sterile 0.85% saline. Susceptibility profiles were collected from *Aeromonas* spp. (*n* = 205), *Edwardsiella* spp. (*n* = 453), *Flavobacterium* spp. (*n* = 800), *Lactococcus* spp. (*n* = 1,215), and *Streptococcus* spp. (*n* = 1,645) isolates across all years and macro-regions.

Two-fold serial dilutions of each antimicrobial were prepared in sterile 96-well microtiter plates to achieve ten distinct concentration gradients spanning the anticipated susceptibility range. Bacterial inocula were standardized to a 0.5 McFarland optical density and subsequently diluted to yield a final concentration of 5 × 10^5^ CFU/mL per well. Incubation was performed at 28 ± 2 °C for 16–20 h under aerobic atmospheric conditions. The minimum inhibitory concentration (MIC) was recorded as the lowest antimicrobial concentration that prevented visible bacterial growth in the microtiter wells. Concurrent quality control validation was performed using the *Staphylococcus aureus* ATCC 29213 reference strain, with results assessed against the quality control ranges specified in the CLSI aquatic-animal documents VET03/VET04 (supplemented by M07 where an aquatic-animal range was unavailable). Phenotypic categorization (susceptible, intermediate, resistant) was assigned using CLSI aquatic-animal interpretive criteria (VET03/VET04) only when a validated breakpoint or epidemiological cut-off value was established for that specific antimicrobial–pathogen combination; for pairings lacking a defined aquatic-animal breakpoint, MIC values were reported numerically without categorical classification, consistent with the CLSI caution that aquatic-animal interpretive criteria are organism-specific and should not be extrapolated across organisms.

### 2.4 Statistical analysis

#### 2.4.1 Definition and calculation of the DPR

The unit of observation was the individual diagnostic record, defined by farm, sampling date, and production stage. For molecular analyses, a record corresponded to a pool of 3–5 fish from the same farm, date, and stage, and was scored positive for a given pathogen when its species-specific qPCR yielded a Ct below 35 (Section 2.3.2). For bacteriological analyses, a record corresponded to an individual clinically affected fish and was scored positive when the target organism was isolated in culture and confirmed to species level by MALDI-TOF MS (Section 2.3.1). Thus, a positive record reflects qPCR detection for molecular records and culture-plus-MALDI-TOF confirmation for bacteriological records; the two modalities were not combined within a single record, and the modality underlying each pathogen’s records is indicated in Table 1. For each pathogen, the DPR was calculated as the number of positive records divided by the total number of records tested for that pathogen, expressed as a proportion (0–1) or percentage, and computed overall and stratified by macro-region, year, and production stage. Because the surveillance program was structured but non-probabilistic, DPR represents apparent prevalence and was not corrected for diagnostic sensitivity or specificity. Because the two modalities have different observational units (pools vs. individual fish), DPR denominators are not strictly comparable across pathogens detected by different modalities, and inter-pathogen comparisons are accordingly interpreted as relative trends rather than as strictly equivalent prevalence estimates (Section 2.1).

**Table 1:** Diagnostic effort and detection positivity rate (DPR) for the seven target pathogens of Nile tilapia (*Oreochromis niloticus*) across the three Brazilian macro-regions (CW, Central-West; NE, Northeast; SE, Southeast) over the three-year study period (2023–2025).

Pathogen	Macro-Region	2023			2024			2025			Total (N°)	Cumulative DPR (%)
		N° Pos	N° samp	DPR %	N° Pos	N° samp	DPR %	N° Pos	N° samp	DPR %		
*Aeromonas* spp.	CW		3	0.0	15	105	14.3		74	0.0	182	8.2
	NE		7	0.0		68	0.0	1	182	0.5	257	0.4
	SE	68	603	11.3	15	691	2.2	13	785	1.7	2079	4.6
Subtotal		**68**	**613**	**11.1**	**30**	**864**	**3.5**	**14**	**1,041**	**1.3**	**2,518**	**4.4**
*Edwardsiella* spp.	CW		8	0.0	18	104	17.3	108	499	21.6	611	20.6
	NE		7	0.0	4	158	2.5	8	296	2.7	461	2.6
	SE	57	1,588	3.6	81	1,316	6.2	418	2,849	14.7	5,753	9.7
Subtotal		**57**	**1,603**	**3.6**	**103**	**1,578**	**6.5**	**534**	**3,644**	**14.7**	**6,825**	**10.2**
*Flavobacterium* spp.	CW		6	0.0	51	83	61.4	18	30	60.0	119	58.0
	NE	3	7	42.9			0.0		13	0.0	20	15.0
	SE	45	366	12.3	61	294	20.7	41	215	19.1	875	16.8
Subtotal		**48**	**379**	**12.7**	**112**	**377**	**29.7**	**59**	**258**	**22.9**	**1,014**	**21.6**
*Francisella orientalis*	CW		22	0.0	58	188	30.9		142	0.0	352	16.5
	NE		39	0.0		136	0.0		12	0.0	187	0.0
	SE	100	1,267	7.9	259	2,227	11.6	251	1989	12.6	5,483	11.1
Subtotal		**100**	**1,328**	**7.5**	**317**	**2,551**	**12.4**	**251**	**2,143**	**11.7**	**6,022**	**11.1**
*ISKNV*	CW	265	659	40.2	234	1840	12.7	512	1,447	35.4	3,946	25.6
	NE	3	1,484	0.2	117	800	14.6	94	528	17.8	2,812	7.6
	SE	1759	6,563	26.8	1858	8,671	21.4	2,375	8,925	26.6	24,159	24.8
Subtotal		**2027**	**8,706**	**23.3**	**2,209**	**11,311**	**19.5**	**2,981**	**10,900**	**27.3**	**30,917**	**23.3**
*Lactococcus* spp.	CW	97	218	44.5	114	492	23.2	125	461	27.1	1,171	28.7
	NE		44	0.0	4	298	1.3	19	320	5.9	662	3.5
	SE	113	860	13.1	370	2,652	14.0	738	3,244	22.7	6,756	18.1
Subtotal		**210**	**1,122**	**18.7**	**488**	**3,442**	**14.2**	**882**	**4,025**	**21.9**	**8,589**	**18.4**
*Streptococcus* spp.	CW	71	192	37.0	85	625	13.6	133	655	20.3	1,472	19.6
	NE		83	0.0	60	405	14.8	66	427	15.5	915	13.8
	SE	169	1966	8.6	534	3,998	13.4	797	4,278	18.6	10,242	14.6
Subtotal		**240**	**2,241**	**10.7**	**679**	**5,028**	**13.5**	**996**	**5,360**	**18.6**	**12,629**	**15.2**
Total		**2,750**	**15,992**	**17.2**	**3,938**	**25,151**	**15.7**	**5,717**	**27,371**	**20.9**	**68,514**	**18.1**

For each pathogen, macro-region, and year, the number of positive samples (N° positive), the number of samples analyzed (N° sample), and the corresponding DPR (%) are shown, together with the regional subtotals, the total number of samples (Total N°), and the cumulative DPR (%).

The cumulative DPR is calculated as the total number of positive samples divided by the total number of samples analyzed, expressed as a percentage. Values in red denote subtotal and total rows. DPR, detection positivity rate.

#### 2.4.2 Modelling of DPR determinants

Factors associated with variation in DPR were assessed at two complementary levels. First, at the pathogen-specific level, a sequential analysis of deviance (Type I) was performed for each pathogen individually using a generalized linear model (GLM) with quasibinomial distribution and logit link, in which year, macro-region, and production stage were added successively as predictors; the significance of each term was evaluated by F-tests on the change in residual deviance. Second, at the assemblage level, all pathogens were analyzed jointly, and six candidate GLM structures of progressive complexity in the interactions among year (df = 3), macro-region (df = 2), and production stage (df = 3) were formulated and compared.

The quasibinomial distribution was selected to model proportion data while accounting for overdispersion, a common feature of positivity-rate datasets, and models were weighted by the number of fish contributing to each record (pool size for molecular records; one for individual bacteriological records) so that records representing more fish carried proportionally greater weight; this replaces the inverse-sample-size weighting used previously, which could down-weight the most informative records. Because diagnostic records are clustered within farms, sampling weeks, and production units, the fixed effects quasibinomial models were complemented by a hierarchical sensitivity analysis in which generalized linear mixed models (binomial, logit link) were fitted with random intercepts for culture site (farm/unit), epidemiological week, and individual record (Supplementary Table S1). The best-supported mixed model retained all three random effects (site, week, and record; AIC = 11,779), and the fixed-effect estimates for pathogen, region, year, and stage remained directionally consistent with the fixed-effects models, indicating that the principal conclusions are robust to farm-level clustering; adding a pathogen × region random term did not improve fit (ΔAIC ≈ + 2; *p* = 1.0). Fixed-effects model selection was based on sequential F-tests on residual deviance and the Akaike Information Criterion (AIC); because AIC is not uniquely defined for quasibinomial fits, it was used only as a relative ranking heuristic alongside F-tests and cross-checked against the likelihood-based binomial mixed models in Supplementary Table S1, retaining the most parsimonious structure that significantly improved fit over the reference model. Collinearity and influential observations were assessed prior to final model selection, model convergence was verified, and the significance threshold was set at α = 0.05. All analyses were performed in R version 4.3.2 32, with generalized linear models fitted using the base stats package and mixed models using lme4.

#### 2.4.3 Time-series analysis

To characterize the temporal dynamics of pathogen DPR and to identify the optimal surveillance frequency, time-series analysis was applied to the epidemiological DPR series of each pathogen. Four temporal aggregation frequencies were evaluated quarterly (freq = 4), monthly (freq = 12), four-monthly (freq = 13), and weekly (freq = 52), with the optimal frequency selected on the basis of the minimum mean squared error (MSE) and mean absolute error (MAE), together with Shapiro–Wilk tests of residual normality.

Stationarity was evaluated using the Augmented Dickey–Fuller (ADF) test, where rejection of the unit-root hypothesis (*p* < 0.05) indicates a stationary process consistent with endemic, equilibrium-phase dynamics. The trend component of each series was extracted by classical time-series decomposition to visualize the underlying directional behavior. Following frequency and stationarity assessment, autoregressive (AR), moving-average (MA), integrated autoregressive moving-average (ARIMA), and white-noise (simple-mean) structures were fitted per pathogen, and the best-supported model was selected by the AIC and the Bayesian Information Criterion (BIC), with residual independence assessed by the Ljung–Box test. All time-series procedures were implemented in R using the forecast (33) and tseries packages (34).

#### 2.4.4 Pathogen co-detection analysis

Patterns of pathogen co-detection were evaluated at the level of the unitary sampling event (defined by farm and date). A presence/absence matrix was constructed for the seven pathogens, each coded as 1 when detected (DP*R* > 0) and 0 otherwise. Pairwise associations between pathogens were first quantified by correlation of their co-detection profiles, and the resulting correlation matrix was displayed as a correlogram, with pathogens ordered by the first principal-component method to group agents with similar co-detection patterns.

To characterize the co-detection structure as a network, an undirected graph was constructed in which nodes represented pathogens and edges represented pairwise co-detection associations. The adjacency matrix was computed from the presence/absence matrix and normalized by the total number of samples to yield the pairwise co-detection frequency; edges below a co-detection frequency of 0.10 were filtered out to focus the analysis on the most consistent associations. Communities of co-detecting pathogens were delineated using the Louvain modularity-optimization algorithm, and the network was visualized with a force-directed layout. Analyses were implemented in R using the corrplot (35) and igraph (36) packages, with community detection by the Louvain algorithm (37), and force-directed visualization (38).

#### 2.4.5 Antimicrobial susceptibility modeling

Given the continuous and positively skewed distribution of MIC values, MIC determinations were first summarized as the representative (median) MIC for each unique combination of pathogen, antibiotic, antibiogram category, and developmental stage; this aggregation yielded 160 combination-level MIC observations, which constituted the modelling units (hence the null residual degrees of freedom of 159 reported in Supplementary Table S6). A generalized linear model (GLM) with Gamma distribution and logarithmic link function was then fitted to these combination-level values to identify the predictors of MIC variation. The Gamma-log parameterization assumes that the variance of MIC is proportional to the square of its mean, an appropriate assumption for right-skewed concentration data. The model considered host developmental stage (fry, juvenile, and adult), pathogen identity, antibiotic (amoxicillin, enrofloxacin, florfenicol, and oxytetracycline), antibiogram category (susceptible, intermediate, and resistant), and their interactions as fixed effects. Because the model was fitted to combination-level summaries rather than to individual isolates, the fixed-effect table reports t statistics and their associated *p*-values (Table 2). Modelling was restricted to the pathogens for which interpretive breakpoints and enough isolates were available (*Aeromonas* spp., *Edwardsiella* spp., *Lactococcus* spp., and *Streptococcus* spp.). *Flavobacterium* spp. and *F. orientalis* were excluded owing to the small number of MIC results in the analyzed dataset.

**Table 2:** Fixed effect estimates from the Gamma GLM (log link) of minimum inhibitory concentration (MIC) for the culturable bacterial pathogens of Nile tilapia.

Predictor	Level	Estimate	Std. error	*t* value	Pr(>|t|)
(Intercept)		3.176	0.762	4.166	0.0001 ***
Stage	Fry	2.327	0.683	3.407	0.0009 ***
	Juvenile	0.162	0.23	0.703	0.483
Pathogen	Edwardsiella	−6.015	1.634	−3.682	0.0003 ***
	Lactococcus	−3.105	1.06	−2.93	0.004 **
	Streptococcus	−3.176	1.407	−2.258	0.0256 *
Antibiotic	ENR	−3.552	0.731	−4.861	< 0.001 ***
	FFC	−2.517	0.764	−3.295	0.0013 **
	OTC	−1.385	0.847	−1.636	0.1042
Antibiogram	Intermediate	0.719	0.505	1.425	0.1565
	Resistant	2.25	0.473	4.755	< 0.001 ***
Pathogen × Antibiotic	Edwardsiella × ENR	5.677	1.607	3.533	0.0006 ***
	Lactococcus × ENR	3.796	1.038	3.658	0.0004 ***
	Streptococcus × ENR	3.62	1.432	2.528	0.0126 *
	Edwardsiella × FFC	5.986	1.642	3.645	0.0004 ***
	Lactococcus × FFC	3.587	1.081	3.318	0.0012 **
	Streptococcus × FFC	3.157	1.43	2.207	0.029 *
	Edwardsiella × OTC	4.805	1.727	2.782	0.0062 **
	Lactococcus × OTC	2.603	1.284	2.027	0.0446 *
	Streptococcus × OTC	2.001	1.68	1.191	0.2358
Pathogen × Antibiogram	Edwardsiella × Intermediate	−0.056	0.835	−0.067	0.9465
	Lactococcus × Intermediate	1.178	0.665	1.772	0.0786
	Streptococcus × Intermediate	0.243	0.629	0.387	0.6997
	Edwardsiella × Resistant	1.12	0.956	1.172	0.2431
	Lactococcus × Resistant	−2.859	1.357	−2.107	0.037 *
	Streptococcus × Resistant	−3.173	1.315	−2.414	0.0172 *

Reference levels: adult (developmental stage), *Aeromonas* spp. (pathogen), amoxicillin (antibiotic), and susceptible (antibiogram category). ENR, enrofloxacin; FFC, florfenicol; OTC, oxytetracycline. Positive estimates denote higher MIC (greater resistance). Significance codes: ***p < 0.001; **p < 0.01; *p < 0.05.

Ten candidate model structures were compared by analysis of deviance and by the Akaike Information Criterion (AIC), spanning progressively more complex combinations of main effects and interactions among stage, pathogen, antibiotic, and antibiogram category. Model selection followed the principle of retaining the most parsimonious structure that significantly improved fit over the simpler reference models, as assessed by likelihood-ratio tests. The best-supported structure was MIC ~ Stage + Pathogen/(Antibiotic × Antibiogram) (AIC = 786), a nested formulation in which the effect of the antibiotic × antibiogram interaction was allowed to vary by pathogen. A marginally lower-AIC alternative was not retained because its additional terms did not significantly improve model fit (*p* = 0.443).

Model coefficients represent the expected change in the logarithm of MIC relative to the reference categories: *Aeromonas* spp. (pathogen), amoxicillin (antibiotic), susceptible (antibiogram category), and adult (developmental stage). Statistical significance was set at *p* < 0.05. In addition to the parametric model, non-parametric group-level comparisons of MIC across antibiogram categories were performed within each pathogen–antibiotic combination using the Kruskal–Wallis test, with pairwise Wilcoxon rank-sum tests for post-hoc comparisons. Analyses were implemented in R version 4.3.2 (39) using the base stats package for the generalized linear model and the Kruskal-Wallis and Wilcoxon rank-sum tests, and the broom package (40) for tidying model outputs.

## 3 Results

### 3.1 Overall diagnostic effort and detection positivity

A total of 26,057 diagnostic samples were analyzed across the three Brazilian macro-regions (CW, NE, and SE) throughout the three-year study period (2023–2025). Because each sample was tested for multiple pathogens, these samples generated 68,514 pathogen-specific diagnostic tests, which constitute the denominators of the DPR analyses reported below. The cumulative overall DPR across all pathogen tests combined was 18.1% (12,405/68,514), remaining relatively stable across years (17.2% in 2023, 15.7% in 2024, and 20.9% in 2025) (Table 1). This aggregate stability, however, concealed marked regional and temporal heterogeneity in the detection of individual pathogens (Figure 1).

**Figure 1:**
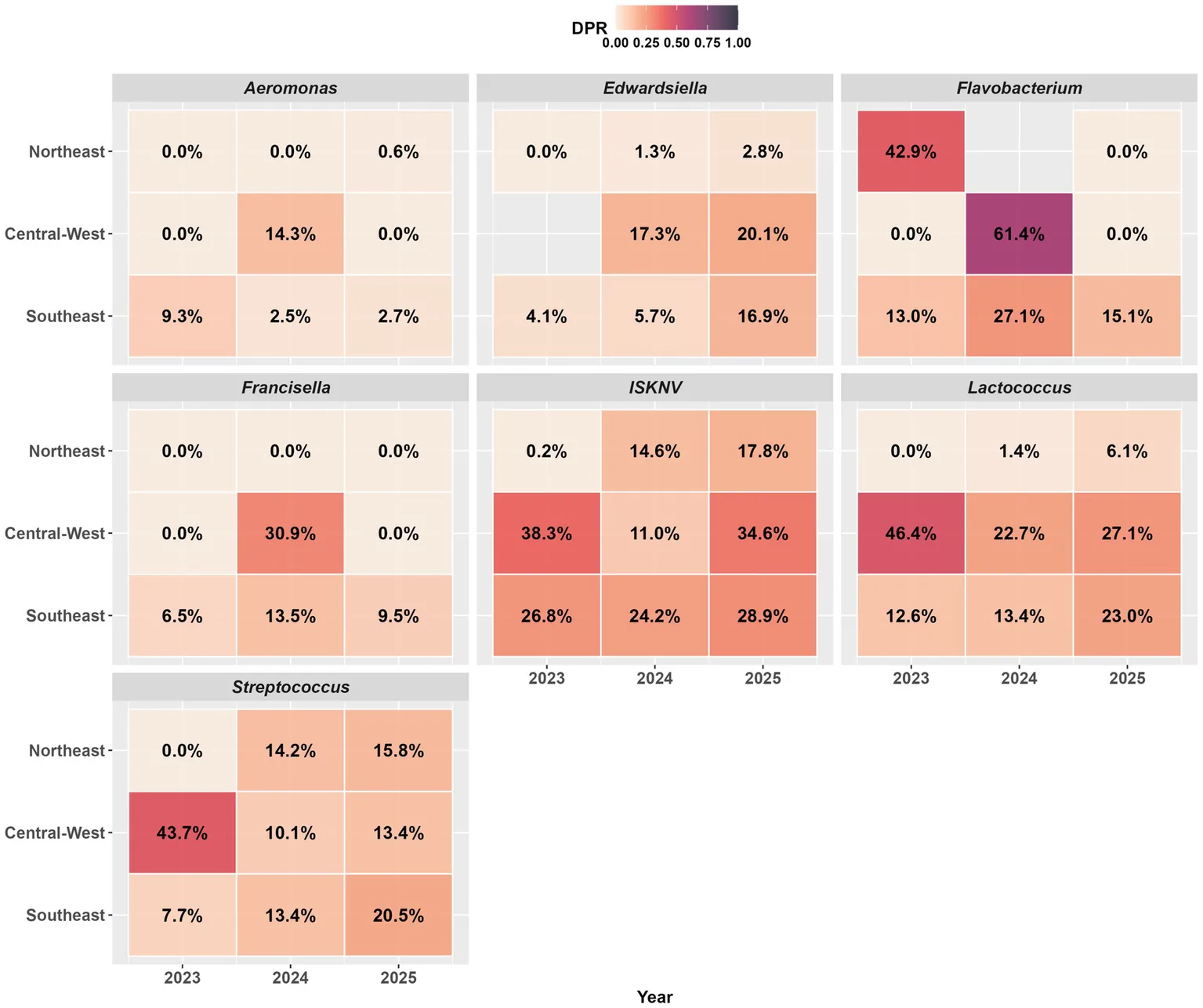
Heat map of the detection positivity rate (DPR) for the seven target pathogens of Nile tilapia across the three Brazilian macro-regions and study years (2023–2025). Each cell represents the DPR (%) for a given pathogen, macro-region (CW, Central-West; NE, Northeast; SE, Southeast), and year, with the numerical value displayed within the cell and colour intensity scaled according to the DPR gradient shown in the legend (0.00–1.00). Empty cells indicate the absence of samples for the corresponding pathogen–region–year combination. The panel arrangement allows direct comparison of temporal trends within each macro-region and of regional differences within each year. This figure is presented as a single panel (a heat map); it does not contain separate sections A and B. The complementary year-on-year dispersion of the underlying DPR values is provided in Supplementary Figure S2.

The diagnostic effort was unevenly distributed among pathogens (Table 1). ISKNV concentrated by far the largest testing volume (30,917 tests; 45.1% of the total), followed by *Streptococcus* spp. (12,629 tests; 18.4%), largely represented by *S. agalactiae* serotype Ib and, to a lesser extent, by *S. agalactiae* serotype III and *S. iniae* (Section 3.1), *Lactococcus* spp. (8,589 tests), *Edwardsiella* spp. (6,825 tests), and *Francisella orientalis* (6,022 tests) (Table 1). Serotype-specific qPCR detected no *S. agalactiae* serotype Ia in any sample, macro-region, or year of the study. By contrast, *Aeromonas* spp. (2,518 tests) and, most notably, *Flavobacterium* spp. (1,014 tests) accounted for the smallest testing bases of the study. Cumulative DPR varied considerably among pathogens and was not proportional to testing effort. *Flavobacterium* spp. yielded the highest cumulative positivity of all agents (21.6%) despite its minimal testing base, whereas *Aeromonas* spp. showed the lowest (4.4%) (Table 1). This dissociation between positivity and testing intensity underscores the need to interpret DPR values in light of their underlying denominators, a caveat developed in the pathogen-specific and spatial analyses below.

### 3.2 Spatiotemporal heterogeneity in pathogen detection

The three most epidemiologically relevant pathogens, ISKNV, *Lactococcus* spp., and *Streptococcus* spp., exhibited stable or moderately increasing cumulative DPRs at the national scale that masked sharply divergent regional dynamics (Table 1; Figure 1; Supplementary Figure S1).

ISKNV accounted for the largest diagnostic effort in the study (30,917 tests; cumulative DPR 23.3%) and the most widespread detection (Table 1; Figure 1). The SE macro-region dominated the test set, accounting for 78% of all ISKNV tests and recording a consistently high positivity rate (21.4–26.8%). The CW macro-region, by contrast, showed a volatile trend, with positivity peaking at 40.2% in 2023, dropping to 12.7% in 2024, and rebounding to 35.4% in 2025 (Figure 1). The most striking pattern was observed in the NE macro-region, where ISKNV was virtually undetectable in 2023 (0.2%; 3/1,484) but increased to 14.6% in 2024 and 17.8% in 2025. Notably, this near-zero baseline derived from the largest single-region testing effort of that year. This emergence is therefore statistically robust and, although the surveillance design is non-probabilistic, is more consistent with true spatial expansion than with intensified detection of an already established infection.

*Lactococcus* spp. (8,589 tests; cumulative DPR 18.4%) was concentrated in the CW and SE macro-regions (Table 1; Figure 1). Positivity peaked in the CW in 2023 (44.5%) and declined thereafter (23.2 and 27.1%) despite a concurrently expanding testing base, indicating a genuine attenuation of positivity rather than a denominator artefact. The SE accounted for most of the testing (79% of all *Lactococcus* tests) and followed an upward temporal pattern (13.1%, 14.0%, and 22.7% across successive years), with the 2025 rebound yielding the highest absolute case count for this pathogen in any region–year (Figure 1).

*Streptococcus* spp. (12,629 tests; cumulative DPR 15.2%) exhibited the most temporally progressive pattern of all pathogens analyzed, with the aggregate DPR increasing steadily across years (10.7%, 13.5%, and 18.6%) against a concurrently expanding testing base, a combination consistent with a genuinely intensifying diagnostic burden rather than a denominator effect (Table 1; Figure 1). The CW peaked early (37.0% in 2023) before stabilizing at a lower plateau, whereas the SE (81% of all *Streptococcus* tests) rose steadily throughout (8.6%, 13.4%, 18.6%).

*Edwardsiella* spp. (6,825 tests; cumulative DPR 10.2%) and *Francisella orientalis* (6,022 tests; cumulative DPR 11.1%) exhibited lower and more spatially restricted detection than the dominant pathogens (Table 1; Figure 1). For both agents, the SE macro-region concentrated the great majority of the testing effort, accounting for 84 and 91% of their respective tests. *Edwardsiella* spp. showed a marked upward trend in the SE (3.6%, 6.2%, and 14.7%), together with a CW increase across the period (0.0%, 17.3%, 21.6%) and NE detection that remained low throughout (≤2.7%). *Francisella orientalis* was almost entirely confined to the SE, where positivity remained stable across years (7.9–12.6%), with the only notable exception being an isolated CW peak in 2024 (30.9%; 58/188) that reverted to zero in 2025; detection was absent from the NE in all three years (0.0%; 0/187). The transient CW peak derived from a small testing base and should be interpreted with caution.

Critically, three of the dominant pathogens shared a pronounced late-emergence signal in the NE macro-region, being undetectable or negligible in 2023 but increasing sharply by 2025 (ISKNV, 17.8%; *Streptococcus* spp., 15.5%; *Lactococcus* spp., 5.9%) (Figure 1). This convergent pattern, also partially observed for *Edwardsiella* spp., is compatible with a shared region-level correlate of increasing pathogen circulation rather than independent, pathogen-specific dynamics; because sampling was non-probabilistic and NE effort expanded over the period, this interpretation is offered as a hypothesis rather than as established causation. Notably, *Edwardsiella* spp. and *Francisella orientalis* did not follow this trajectory, remaining spatially anchored to the established SE production core throughout the study period. The year-on-year dispersion of individual DPR values underlying these trends is shown in Supplementary Figure S2.

### 3.3 Fine-scale spatial distribution of pathogen detection

Beyond the macro-regional aggregates, the georeferenced distribution of positive detections showed that pathogen circulation was spatially concentrated rather than uniformly distributed across the Brazilian territory (Figure 2). Detection foci clustered predominantly along the SE–CW axis. The NE signal was comparatively sparse and appeared later, consistent with the temporal trends quantified at the macro-regional scale (Table 1; Figure 1).

**Figure 2:**
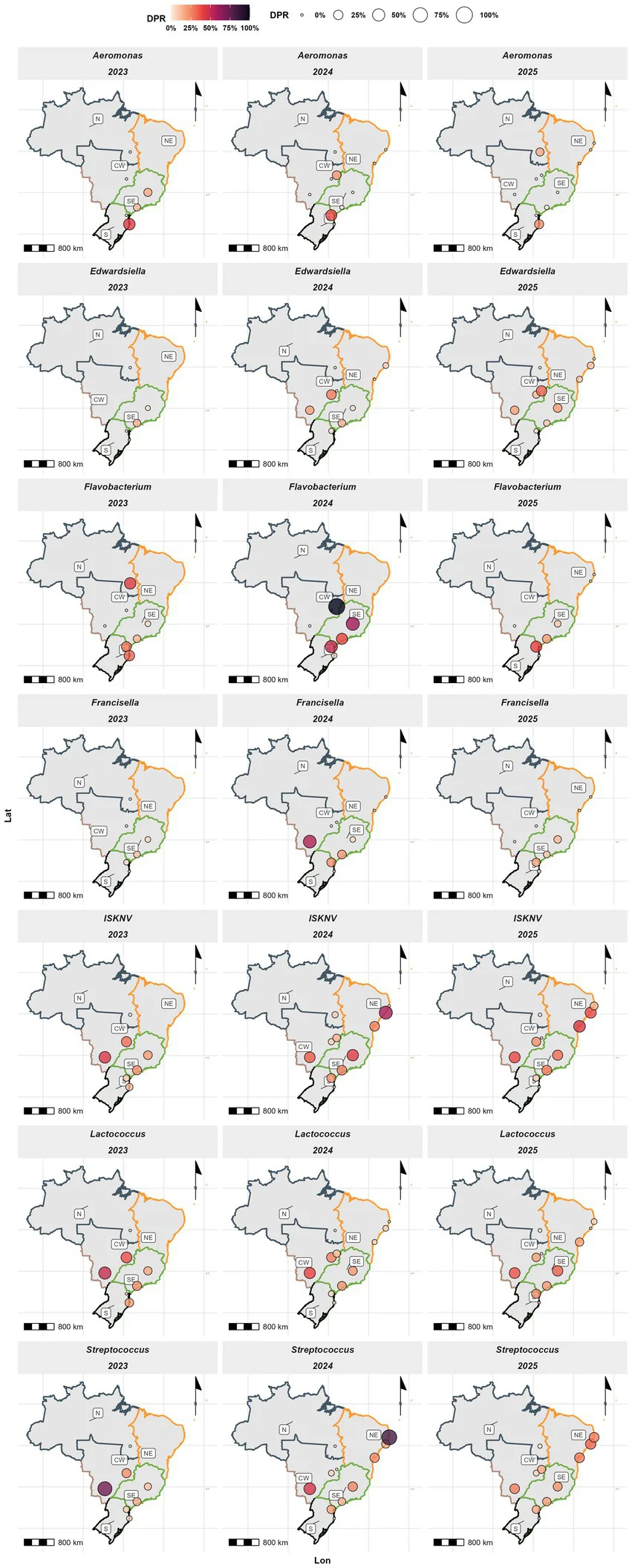
Georeferenced distribution of positive detections for the seven target pathogens of Nile tilapia across Brazil over the study period (2023–2025). Panels are arranged sequentially by pathogen and, within each pathogen, by consecutive year (2023, 2024, and 2025), as indicated by the label above each map. Each point denotes a detection locality; point size is proportional to the number of samples analyzed and color intensity reflects the detection positivity rate (DPR), according to the scales shown in the legend. The three Brazilian macro-regions are delineated on the base maps (CW, Central-West; NE, Northeast; SE, Southeast). The sequential arrangement allows the year-on-year progression of each pathogen to be followed within its block of three panels and illustrates the overall expansion of detection foci from a SE–CW core toward the NE over the study period.

The panel arrangement, in which each pathogen is displayed across the three consecutive study years, allowed the temporal progression of each agent to be tracked at the locality level (Figure 2). For most pathogens, the 2023 panels showed detection confined to a limited number of foci in the SE and CW macro-regions. The 2024 and 2025 panels displayed both a greater number of positive localities and a northward extension of the detection footprint. This spatial expansion was most evident for ISKNV, *Streptococcus* spp., and *Lactococcus* spp. Their 2025 panels showed the densest and most widely dispersed arrangement of positive points, including new NE foci that were absent or negligible in 2023. The progressive appearance of georeferenced positives in the NE provides independent spatial corroboration of the late-emergence pattern inferred from the regional DPR trends (Figure 1). This finding supports genuine geographic spread rather than a detection artefact.

The maps further illustrate the interplay between sampling intensity (point size) and positivity (color intensity) (Figure 2). High-DPR foci associated with small sampling volumes appeared as intensely colored but small points, as observed for *Flavobacterium* spp. in the CW macro-region. This pattern visually reinforces the disproportion between elevated positivity and limited sampling effort noted above (Table 1). By contrast, the SE concentrated numerous large points of moderate-to-high intensity across all years. This distribution reflects both its dominant sampling base and its role as the sustained epidemiological core for most pathogens.

In contrast to the expanding pathogens, the maps confirmed the spatially restricted distribution of *Edwardsiella* spp. and *Francisella orientalis* (Figure 2). For both agents, detection foci were concentrated in the SE macro-region across all three years. *Edwardsiella* spp. showed a predominantly SE-based arrangement of foci, with a limited CW signal appearing in 2024 that did not expand in subsequent panels and no sustained NE foci. *Francisella orientalis* showed one of the most spatially compact distributions of all pathogens analyzed, with foci remaining centered on the SE throughout. Neither agent showed evidence of the progressive NE expansion recorded for the dominant pathogens.

Overall, the spatial analysis indicates that pathogen detection in Brazilian Nile tilapia aquaculture is characterized by a persistent SE–CW core and a temporally expanding NE frontier. This pattern was shared across several taxonomically and biologically distinct agents, suggesting a common region-level driver of increasing pathogen circulation. Nevertheless, this expansion was not universal: *Edwardsiella* spp. and *Francisella orientalis* remained confined to the established SE production core, reinforcing the distinction between pathogens undergoing geographic expansion and those with a stable, spatially restricted distribution (Table 1; Figure 2).

### 3.4 Effect of developmental stage on pathogen detection

The contribution of host developmental stage to variation in detection positivity was assessed at two levels. When each pathogen was modelled separately by sequential deviance analysis, developmental stage (Fase) significantly improved model fit for *Aeromonas* spp. (*p* < 2.2 × 10^−16^), *Streptococcus* spp. (*p* = 1.1 × 10^−6^), and *Lactococcus* spp. (*p* = 0.009) once region and year had been accounted for, but not for the remaining four agents (all *p* > 0.05). Year contributed significantly for *Aeromonas* spp. (*p* = 1.9 × 10^−5^), *Francisella orientalis* (*p* = 0.011), and *Lactococcus* spp. (*p* = 0.005), whereas macro-region did not significantly improve fit for any pathogen in these individual models (all *p* > 0.05). At the pathogen-specific level, therefore, the determinants of DPR were heterogeneous, with developmental stage influencing positivity in some agents but not others.

When all pathogens were analyzed jointly, a set of nested fixed-effects generalized linear models (GLM; quasibinomial, logit link) was compared by sequential analysis of deviance (Supplementary Table S2). Among these, the additive structure DP*R* ~ Pathogen + Year + Macro-Region + Stage (Model 3) was retained as the interpretable fixed-effects model; it includes developmental stage as an explanatory term, indicating that stage contributes measurable explanatory power at the level of the pathogen assemblage. Because the fixed-effects GLM does not account for the hierarchical structure of the data, it was complemented by a generalized linear mixed-model (GLMM) analysis (Tables 3, 4; model selection in Supplementary Table S3), which constitutes the primary inferential model for the determinants of DPR.

**Table 3:** Analysis of variance (Type II Wald chi-square) of the selected generalized linear mixed model (GLMM; binomial, logit link), evaluating the fixed effects of year, macro-region, and host developmental stage on the probability of detection for each pathogen, together with the variance and standard deviation of the random effects by group (Site, epidemiological week).

Pathogen	Predictor	Sum Sq	Mean Sq	*F* value	Chisq	df	Pr(>Chisq)	Random group	Variance	Std. dev.
*Aeromonas*	Año	17.768	8.884	8.884	14.388	2	7.5e-04 ***	Site	18.336	4.282
	Region	0.128	0.064	0.064	0.254	2	0.8806	Semana	4.297	2.073
*Edwardsiella*	Año	90.034	45.017	45.017	89.44	2	< 2.2e-16 ***	Site	27.53	5.247
	Region	1.798	0.899	0.899	1.686	2	0.4304	Semana	2.07	1.439
*Francisella*	Año	37.024	18.512	18.512	37.399	2	7.6e-09 ***	Site	184.52	13.584
	Region	0.022	0.011	0.011	0.262	2	0.8774	Semana	61.66	7.85
*Flavobacterium*	Año	11.873	5.937	5.937	11.941	2	0.0026 **	Site	107.297	10.358
	Region	0.038	0.019	0.019	0.259	2	0.8786	Semana	1.227	1.108
*Lactococcus*	Region	2.82	1.41	1.412	4.654	2	0.0976	Site	9.852	3.139
	Año	29.93	14.96	14.963	55.932	2	7.2e-13 ***	Semana	10.52	3.243
	Fase	1518.78	759.39	759.39	1508.55	2	< 2.2e-16 ***			
ISKNV	Region	4.52	2.258	2.258	4.072	2	0.1305	Site	23.809	4.879
	Año	69.65	34.823	34.823	61.398	2	4.6e-14 ***	Semana	1.235	1.111
	Fase	320.7	160.352	160.352	322.015	2	< 2.2e-16 ***			
*Streptococcus*	Region	4.33	2.165	2.165	4.034	2	0.133	Site	8.033	2.834
	Año	1.568	0.784	0.784	1.552	2	0.4602	Semana	2.954	1.719

Sum Sq, sum of squares; Mean Sq, mean square; *F* value, F statistic; Chisq, chi-square; df, degrees of freedom; Pr(>Chisq), *p*-value. Significance codes: ***p < 0.001; **p < 0.01; *p < 0.05.

**Table 4:** Odds ratios (OR) and 95% confidence intervals (CI) of the fixed effects, and variances of the random effects, from the per-pathogen generalized linear mixed models for pathogen detection in Nile tilapia. σ^2^, residual variance; τ00 Site, between-site variance; τ00 Semana, between-epidemiological-week variance; ICC, intraclass correlation coefficient; N, number of levels; Marginal R^2^, variance explained by fixed effects; Conditional R^2^, variance explained by fixed and random effects.

Pathogen	Predictor	OR	95% CI	p	Random effect	Value
*Aeromonas*	(Intercept)	0	0–0.25	0.013 *	σ2	0.88
	2024	0.08	0.01–0.6	0.014 *	τ00 Site	18.34
	2025	0.01	0–0.11	< 0.001 ***	τ00 Semana	4.3
	Central-West	1.25	0–1646.1	0.951	ICC	0.96
	Southeast	3.03	0.02–486.02	0.669	N Semana	40
					N Site	56
					Observations	520
					Marginal R2	0.152
					Conditional R2	0.968
*Edwardsiella*	(Intercept)	0	0–0.01	< 0.001 ***	σ2	0.75
	2024	3.82	2.07–7.04	< 0.001 ***	τ00 Site	27.53
	2025	10.84	6.07–19.38	< 0.001 ***	τ00 Semana	2.07
	Central-West	28.03	0.05–17164.45	0.309	ICC	0.98
	Southeast	1.07	0.01–95.36	0.978	N Semana	44
					N Site	77
					Observations	1,273
					Marginal R2	0.064
					Conditional R2	0.977
*Flavobacterium*	(Intercept)	0	0–0.15	0.016 *	σ2	0.73
	2024	6.74	2.28–19.89	0.001 **	τ00 Site	107.3
	2025	3.93	1.2–12.82	0.023 *	τ00 Semana	1.23
	Central-West	0.3	0–6815.27	0.813	ICC	0.99
	Southeast	1.73	0–6434.13	0.896	N Semana	28
					N Site	36
					Observations	172
					Marginal R2	0.007
					Conditional R2	0.993
*Francisella*	(Intercept)	0	0–0.15	0.016 *	σ2	0.73
	2024	6.74	2.28–19.89	0.001 **	τ00 Site	107.3
	2025	3.93	1.2–12.82	0.023 *	τ00 Semana	1.23
	Central-West	0.3	0–6815.27	0.813	ICC	0.99
	Southeast	1.73	0–6434.13	0.896	N Semana	28
					N Site	36
					Observations	172
					Marginal R2	0.007
					Conditional R2	0.993
ISKNV	(Intercept)	0.01	0–0.3	0.006 **	σ2	1
	Central-West	3.13	0.05–190.23	0.586	τ00 Site	28.57
	Southeast	0.13	0–3.5	0.226	τ00 Semana	1.24
	2024	0.93	0.82–1.06	0.289	ICC	0.97
	2025	1.31	1.14–1.5	< 0.001 ***	N Semana	53
	Fry	0.04	0.03–0.07	< 0.001 ***	N Site	119
	Juvenile	1.44	1.23–1.67	< 0.001 ***	Observations	8,098
					Marginal R2	0.063
					Conditional R2	0.97
*Lactococcus*	(Intercept)	0.01	0–0.08	< 0.001 ***	σ2	0.54
	Central-West	34.32	1.36–867.61	0.032 *	τ00 Site	9.85
	Southeast	5.34	0.44–65.11	0.189	τ00 Semana	10.52
	2024	0.54	0.39–0.73	< 0.001 ***	ICC	0.97
	2025	0.88	0.64–1.2	0.414	N Semana	51
	Fry	0.01	0–0.01	< 0.001 ***	N Site	94
	Juvenile	0.06	0.05–0.07	< 0.001 ***	Observations	2,613
					Marginal R2	0.135
					Conditional R2	0.978
*Streptococcus*	(Intercept)	0.09	0.02–0.57	0.01 *	σ2	0.91
	Central-West	0.24	0.02–3.14	0.279	τ00 Site	8.03
	Southeast	0.15	0.02–0.96	0.045 *	τ00 Semana	2.95
	2024	1.15	0.85–1.56	0.374	ICC	0.92
	2025	1.05	0.77–1.42	0.775	N Semana	51
					N Site	107
					Observations	2,933
					Marginal R2	0.029
					Conditional R2	0.926

To account for repeated sampling within farms and weeks, the combined model was refitted as a binomial GLMM with random intercepts for culture site (farm/unit), epidemiological week, and an observation-level random effect (ID). Among the candidate random-effect structures compared, the model retaining site, week, and ID was best supported (lowest AIC = 11,779; Supplementary Table S3). In this selected GLMM, a Type II Wald analysis of variance (Table 3) confirmed that developmental stage was a strong and highly significant determinant of detection for *Lactococcus* spp. and ISKNV (both *p* < 2.2 × 10^−16^) and that year was significant for most pathogens, whereas macro-region was generally non-significant once site was modelled as a random effect; the between-site variance was substantial for every pathogen, confirming strong farm-level clustering. The corresponding odds ratios and random-effect variances are reported in Table 4. The pathogen, region, year, and stage effects remained directionally consistent with the fixed-effects models, confirming that the reported determinants of DPR are robust to farm-level clustering and are not artefacts of pseudoreplication.

The two approaches are therefore complementary rather than contradictory. In the pathogen-specific models, developmental stage was a significant determinant of DPR for the agents with the largest and most balanced stage-specific sample sizes (*Aeromonas* spp., *Streptococcus* spp., and *Lactococcus* spp.), but not for the agents with sparser or more uneven stage representation, evident in the wide DPR ranges spanning much of the 0–1 scale in several groups (Supplementary Figures S3, S4). The combined model, which aggregated observations across all pathogens (residual df > 16,000; Table 4), retained a single, assemblage-level stage term, indicating a modest but consistent overall stage effect once all agents are pooled. Taken together, these results indicate that developmental stage exerts a measurable influence on detection positivity that is strong for some individual pathogens and, at the assemblage level, resolves into a weak but consistent overall signal. The stage-related differences apparent in the raw data (Supplementary Figures S3, S4) should be interpreted considering the uneven stage-specific sample sizes and confirmed in analyses based on larger and more balanced designs.

### 3.5 Temporal dynamics and predictive modelling of pathogen detection

The temporal behavior of each pathogen’s DPR series was first characterized by testing for stationarity using the Augmented Dickey–Fuller (ADF) test. Two of the seven series failed to reject the unit-root hypothesis. For *Streptococcus* spp. (ADF statistic = −2.75; *p* = 0.267), non-stationarity was accompanied by a sustained year-on-year increase against an expanding testing base and is interpreted, cautiously, as consistent with genuine geographic expansion. For *Flavobacterium* spp. (ADF statistic = −2.10; *p* = 0.535), by contrast, non-stationarity arose from abrupt shifts generated by very small period-specific denominators and is regarded as an artefact of sampling instability rather than evidence of directional epidemiological change.

We note that neither ADF *p*-value is statistically decisive on its own. The stationarity classification is therefore treated as a descriptive summary of series behavior rather than as independent proof of endemic establishment. These ADF results should additionally be interpreted with caution because the underlying DPR series may not fully satisfy the test assumptions: sampling intervals were not strictly regular (periods with few or no submissions occur for some pathogens), and the variance of the proportion-based DPR is not homogeneous across the series, being inflated at the low denominators characteristic of sparsely sampled periods. The stationarity labels are therefore best regarded as indicative of broad temporal behavior rather than as formal unit-root inference. The remaining five pathogens (*Aeromonas* spp., *Edwardsiella* spp., *Francisella orientalis*, ISKNV, and *Lactococcus* spp.) yielded stationary series (ADF *p* ≤ 0.05; Table 5), indicating stable temporal means suitable for direct autoregressive modelling; candidate autoregressive (ARIMA) structures for each series are compared in Supplementary Table S4.

**Table 5:** Descriptive statistics and stationarity of the pathogen-specific detection positivity rate (DPR) time series, assessed by the Augmented Dickey–Fuller (ADF) test. N obs., number of observations; SD, standard deviation; CV, coefficient of variation.

Pathogen	N obs	Mean DPR	SD	CV	Min	Max	ADF stat.	ADF p	Stationary
*Aeromonas*	61	0.074	0.1759	2.3781	0	1	−5.2743	0.01	Yes
*Edwardsiella*	79	0.0729	0.1193	1.6375	0	0.5	−3.5641	0.042	Yes
*Flavobacterium*	41	0.1976	0.2621	1.3263	0	1	−2.0969	0.5354	**No**
*Francisella*	86	0.0889	0.213	2.3957	0	1	−3.6465	0.0341	Yes
ISKNV	131	0.3059	0.2974	0.9722	0	1	−4.4581	0.01	Yes
*Lactococcus*	99	0.2084	0.3026	1.4523	0	1	−4.4609	0.01	Yes
*Streptococcus*	101	0.1594	0.2163	1.3572	0	1	−2.7461	0.2674	**No**

A series was classified as stationary when the ADF *p*-value was ≤ 0.05. Non-stationary series are shown in bold.

The observed DPR series and their extracted trend components are shown for each pathogen in Figure 3. Consistent with the stationarity results, most series fluctuated around a stable trend without directional drift: ISKNV, *Lactococcus* spp., and *Edwardsiella* spp. displayed frequent short-term oscillations around a relatively constant mean, whereas the trend component of *Streptococcus* spp. showed a sustained directional pattern, in agreement with its non-stationary classification.

**Figure 3:**
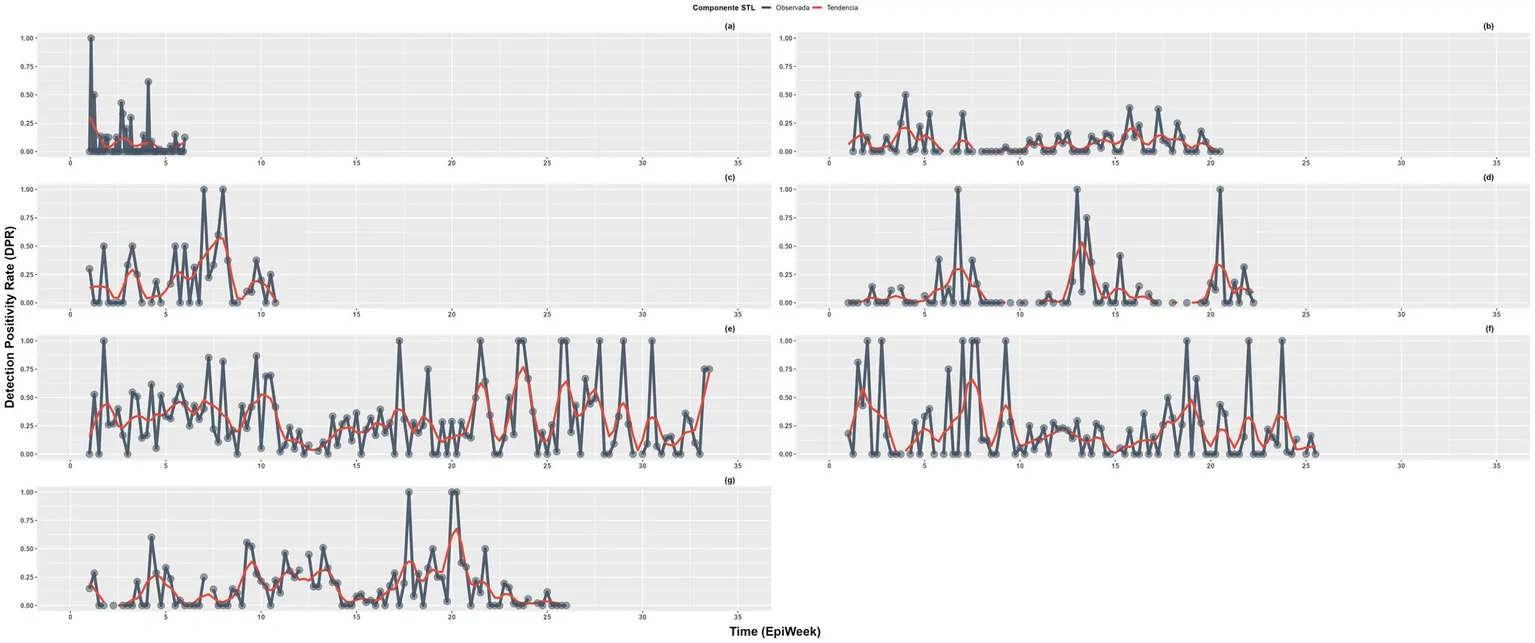
Observed detection positivity rate (DPR) time series and extracted trend components for the seven target pathogens of Nile tilapia over the study period. For each pathogen, the observed DPR (dark line and points) is shown together with its smoothed trend component (red line) across consecutive sampling periods (x-axis). The number of sampling periods differs among pathogens according to their respective sampling frequencies. Most series fluctuate around a stable trend, consistent with the stationarity results, whereas Streptococcus spp. displays a directional trend consistent with its non-stationary classification.

*F. orientalis* yielded a stationary series (ADF statistic = −3.65; *p* = 0.034) across 86 observation periods. Its coefficient of variation was the highest of the seven agents (CV = 2.40), and the series spanned the full 0.00–1.00 range. This combination of high relative variability around a stable, low mean is consistent with sporadic, episodic detection rather than with directional temporal change, and contrasts with the sustained drift observed for *Streptococcus* spp. (Table 5).

The variability of the series differed markedly among pathogens. *F. orientalis* and *Aeromonas* spp. showed the highest coefficients of variation (CV = 2.40 and 2.38, respectively), reflecting sporadic, low-baseline detection, whereas ISKNV and *Lactococcus* spp. exhibited lower relative variability (CV = 0.97 and 1.45) around higher mean DPR values (30.6 and 20.8%), consistent with their more sustained circulation.

For each pathogen, candidate time-series models [autoregressive AR(1,0,0), moving-average MA(0,0,1), and simple-mean structures] were compared by information criteria. Model selection favored parsimonious structures across pathogens, with residual variance scaling with pathogen burden: it was lowest for low-prevalence agents (σ^2^ = 0.12 for Edwardsiella spp. and 0.18 for Aeromonas spp.) and highest for the most widely detected agents (σ^2^ = 0.30 for ISKNV and Lactococcus spp.).

Predictive performance was further evaluated across four temporal aggregation frequencies (quarterly, monthly, four-monthly, and weekly), each applied to the pathogen-specific series (*N* = 41–131 observation periods depending on the pathogen). Prediction error was lowest at the quarterly scale for every pathogen, with mean absolute errors ranging from 0.068 (*Edwardsiella* spp.) to 0.181 (*Lactococcus* spp.). As expected, higher-burden pathogens showed larger absolute errors, mirroring their greater DPR variance. Notably, the Shapiro–Wilk test indicated non-normal residual distributions for most pathogens and frequencies (Supplementary Table S5). This departure from normality should be considered when interpreting the prediction intervals and supports the use of distribution-robust or non-parametric approaches in future forecasting work.

### 3.6 Pathogen co-detection patterns

Pairwise associations between pathogens were examined to assess the extent of co-detection across samples (Figure 4). All pairwise correlations were weak, ranging from −0.02 to 0.12. The strongest positive associations, although modest, were observed among the three dominant pathogens, *Lactococcus* spp.–ISKNV (*r* = 0.12), *Lactococcus* spp.–*Streptococcus* spp. (*r* = 0.11), and *Edwardsiella* spp.–ISKNV (*r* = 0.09), together with a comparable association between *Edwardsiella* spp. and *Flavobacterium* spp. (*r* = 0.11). No negative associations of appreciable magnitude were detected (Figure 4).

**Figure 4:**
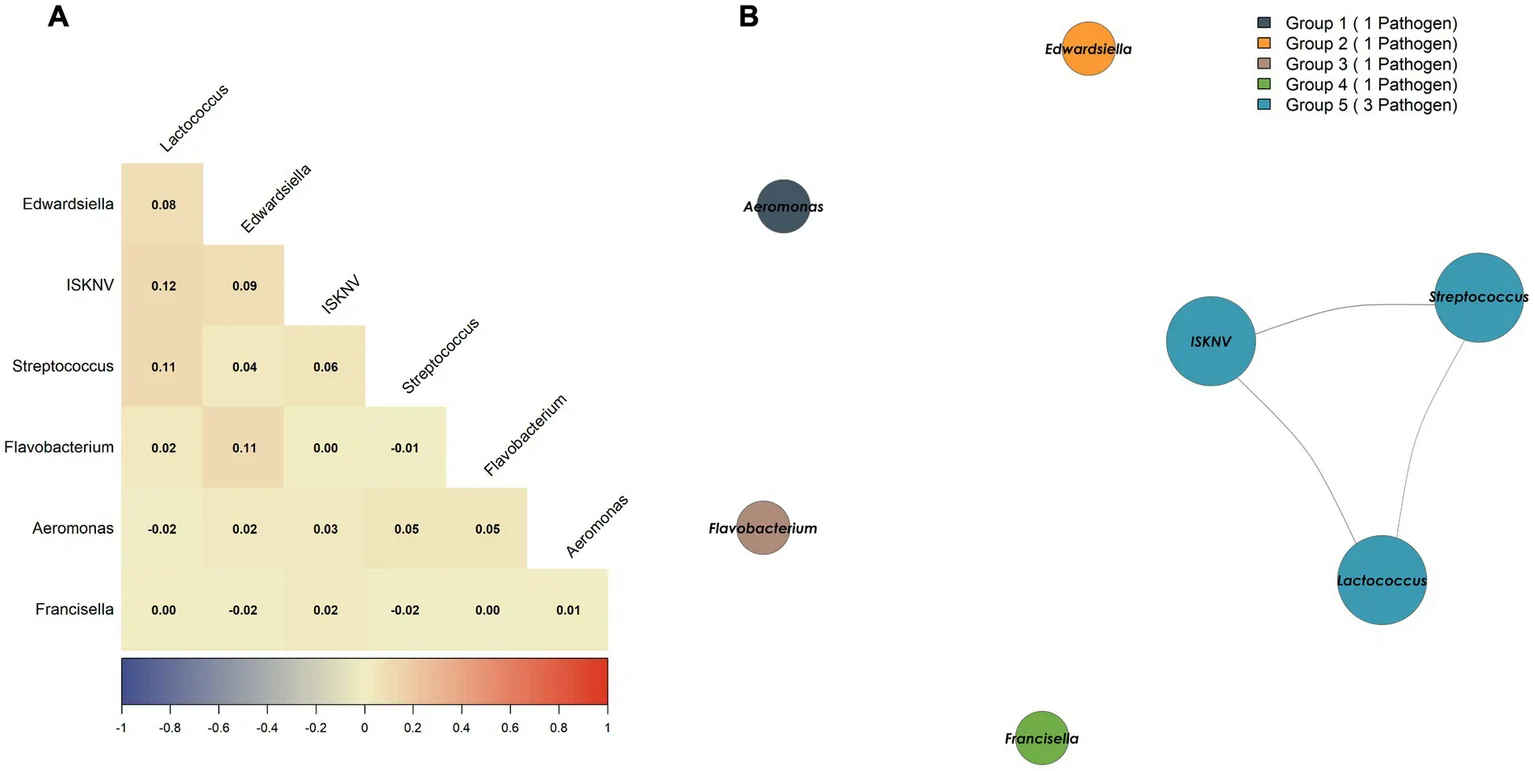
Pathogen co-infection analysis. **(A)** Pairwise co-detection correlation matrix for the seven target pathogens of Nile tilapia. Each cell shows the correlation coefficient between the co-detection of two pathogens across samples; color intensity reflects the magnitude and direction of the correlation according to the scale shown (−1 to +1). All observed associations were weak (range −0.02 to 0.12). **(B)** Co-detection network of the seven target pathogens of Nile tilapia, with communities identified by the Louvain algorithm. Nodes represent pathogens and edges represent pairwise co-detection associations; node color denotes community membership. Four pathogens formed single-member, unconnected groups, whereas ISKNV, *Streptococcus* spp., and *Lactococcus* spp. formed the only multi-member community.

Community detection applied to the co-detection network (Louvain algorithm) resolved the seven pathogens into five groups (Figure 4). Four of these consisted of a single, unconnected pathogen (*Aeromonas* spp., *Edwardsiella* spp., *Flavobacterium* spp., and *Francisella orientalis*), indicating that these agents were detected largely independently of the others. Network centrality metrics corroborated this structure, identifying ISKNV, Streptococcus spp., and Lactococcus spp. as the only influential nodes (non-zero degree and eigenvector centrality), whereas the remaining four agents were peripheral (Table 6). The only multi-member community comprised three pathogens, ISKNV, *Streptococcus* spp., and *Lactococcus* spp., which formed an interconnected module (Figure 4). This module corresponds precisely to the three most widely detected and geographically expanding pathogens identified in the preceding analyses.

**Table 6:** Centrality metrics of the pathogen co-detection network, classifying the seven target pathogens into influential nodes (ISKNV, Streptococcus spp., Lactococcus spp.) and peripheral nodes (Aeromonas spp., Edwardsiella spp., Flavobacterium spp., Francisella orientalis).

Pathogen	Degree	Degree (norm.)	Betweenness	Betw. (norm.)	Closeness	Close (norm.)	Eigenvector
ISKNV	**2**	0.333	0	0	3.271	6.542	**1**
*Streptococcus*	**2**	0.333	0	0	3.558	7.115	**0.953**
*Lactococcus*	**2**	0.333	0	0	4.128	8.257	**0.853**
*Aeromonas*	0	0	0	0	NaN	NaN	0
*Edwardsiella*	0	0	0	0	NaN	NaN	0
*Flavobacterium*	0	0	0	0	NaN	NaN	0
*Francisella*	0	0	0	0	NaN	NaN	0

Degree, number of co-detection edges; Betweenness, number of shortest paths passing through a node; Closeness, inverse mean distance to other nodes; Eigenvector, influence weighted by neighbour influence; norm., normalized value. NaN denotes an isolated node with undefined closeness. Influential nodes are shown in bold.

Overall, these results indicate that pathogen circulation was predominantly independent, with no evidence of strong pairwise co-detection. The single exception was a weak association among the three dominant pathogens (all *r* ≤ 0.12), whose tendency to co-occur is most plausibly explained by shared exposure to the intensive production environments and regions in which all three circulate, rather than by direct biological interaction. Given the very small magnitude of these correlations, the resulting three-pathogen module should be regarded as a weak, hypothesis-generating signal that does not, on its own, establish biologically meaningful co-infection structure; it is nonetheless consistent with the convergent spatiotemporal distributions of these agents (Sections 3.2–3.3) and motivates targeted, longitudinal co-infection studies.

### 3.7 Antimicrobial resistance patterns and minimum inhibitory concentrations

Antimicrobial susceptibility was characterized through minimum inhibitory concentration (MIC) values for the culturable bacterial pathogens, modelled as a function of host developmental stage (fry, juvenile, and adult), pathogen identity, antibiotic (amoxicillin, enrofloxacin, florfenicol, and oxytetracycline), antibiogram category (susceptible, intermediate, and resistant), and their interactions.

Sequential deviance analysis of the combination-level MIC values (160 observations; null residual df = 159; Supplementary Table S6) showed that MIC was significantly influenced by all four main effects — developmental stage (*p* = 0.012), pathogen identity (*p* = 0.001), antibiotic (*p* < 0.001), and antibiogram category (*p* < 0.001) — as well as by the pathogen × antibiogram interaction (*p* = 0.047), whereas the pathogen × antibiotic interaction was only marginal (*p* = 0.061) (Supplementary Table S6). Among the ten candidate structures compared by information criteria, model selection favored MIC ~ Stage + Pathogen/(Antibiotic × Antibiogram) (Model 6), which combined a low AIC (786) with a significant improvement in fit over the simpler reference structures (likelihood-ratio test, *p* < 0.001; Supplementary Table S7). This nested formulation, in which the antibiotic × antibiogram interaction was allowed to vary by pathogen, was retained over one marginally lower-AIC but non-significant alternative (Model 7; *p* = 0.443), and over the main-effect models (Models 1–5; AIC ≈ 1,461–1,480), confirming that both pathogen nesting and the antibiotic × antibiogram interaction were necessary to capture MIC variation adequately.

The selected model quantified the direction and magnitude of each factor (Table 2). Developmental stage was a significant determinant of MIC: relative to the adult reference stage, the fry stage was associated with markedly higher MIC values (estimate = +2.327; *p* < 0.001), indicating elevated resistance in the earliest life stage, whereas the juvenile stage did not differ significantly from adults (estimate = +0.162; *p* = 0.483). Pathogen identity showed strong and consistent effects: relative to the reference pathogen (*Aeromonas* spp.), *Edwardsiella* spp. (estimate = −6.015; *p* < 0.001), *Streptococcus* spp. (estimate = −3.176; *p* = 0.026), and *Lactococcus* spp. (estimate = −3.105; *p* = 0.004) were all associated with significantly lower MIC values. This identifies *Aeromonas* spp. as the pathogen with the highest baseline MIC and the remaining agents as comparatively more susceptible.

The antibiotic tested was likewise a strong determinant of MIC (Table 2). Relative to amoxicillin, both enrofloxacin (estimate = −3.552; *p* < 0.001) and florfenicol (estimate = −2.517; *p* = 0.001) were associated with significantly lower MIC values, whereas oxytetracycline showed a lower but non-significant effect (estimate = −1.385; *p* = 0.104). As expected, isolates classified as resistant on the antibiogram exhibited significantly higher MIC values than susceptible isolates (estimate = +2.25; *p* < 0.001), confirming the concordance between categorical antibiogram classification and quantitative MIC measurement, whereas the intermediate category did not differ significantly from susceptible (*p* = 0.155).

The significant pathogen × antibiotic interactions carried the most direct interpretive weight, indicating that the relationship between MIC and antibiotic differed among pathogens (Table 2). *Edwardsiella* spp. and *Lactococcus* spp. showed significantly elevated MIC values in combination with enrofloxacin (estimates = +5.677 and +3.796, respectively; both *p* < 0.001) and florfenicol (estimates = +5.986 and +3.587; *p* < 0.001 and *p* = 0.001), and *Edwardsiella* spp. also with oxytetracycline (estimate = +4.805; *p* = 0.006); *Streptococcus* spp. showed a comparable, significant elevation with enrofloxacin (estimate = +3.620; *p* = 0.012). These pathogen-specific interactions demonstrate that susceptibility to an individual antibiotic cannot be generalized across pathogens, so that the low baseline MICs observed for some agents do not translate uniformly across all compounds. In contrast, the pathogen × antibiogram interactions were largely non-significant, with the exceptions of *Lactococcus* spp. and *Streptococcus* spp. in the resistant category (estimates = −2.859 and −3.173; *p* = 0.037 and *p* = 0.017), indicating that the MIC increment associated with phenotypic resistance was somewhat attenuated for these two streptococcal agents relative to the reference pathogen.

The distribution of MIC values across antibiogram categories is shown for each pathogen and antibiotic in Figure 5. In most pathogen–antibiotic combinations, MIC increased significantly from susceptible to resistant categories (Kruskal–Wallis test; panel-specific *p*-values shown in Figure 5), confirming at the level of individual combinations the overall concordance quantified in the model. The separation was most pronounced for the combinations involving *Aeromonas* spp., consistent with its high baseline MIC, and least consistent for combinations in which resistant isolates were scarce or absent, such as amoxicillin against *Streptococcus* spp. (*p* = 0.12) and oxytetracycline against *Edwardsiella* spp. (*p* = 0.12).

**Figure 5:**
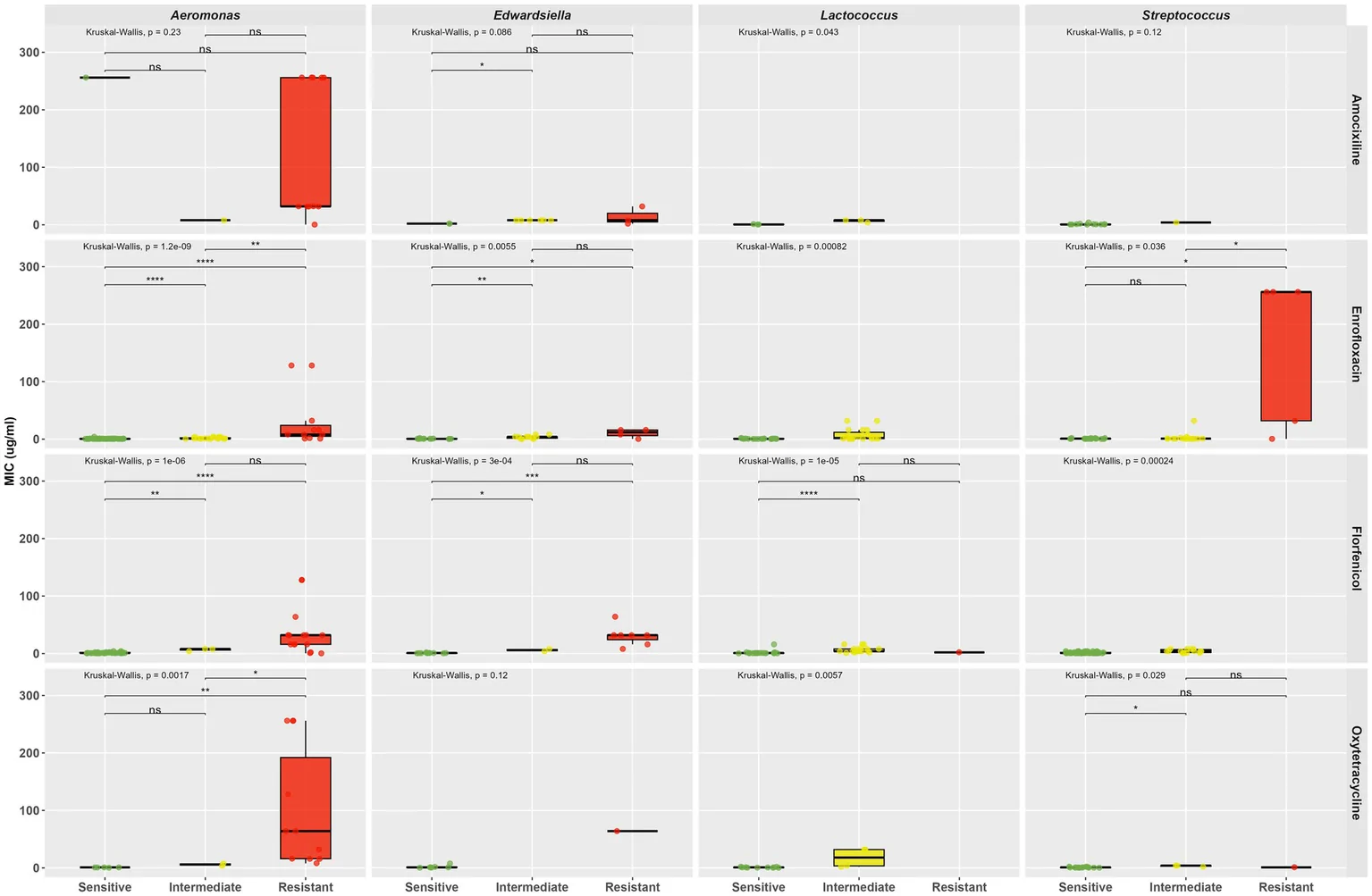
Distribution of minimum inhibitory concentration (MIC, μg/mL) values across antibiogram susceptibility categories (Sensitive, Intermediate, Resistant) for four bacterial pathogens of Nile tilapia (columns: *Aeromonas* spp., *Edwardsiella* spp., *Lactococcus* spp., and *Streptococcus* spp.) and four antimicrobials (rows: amoxicillin, enrofloxacin, florfenicol, and oxytetracycline). Within each panel, box plots show the median and interquartile range of MIC by category, with individual observations overlaid as jittered points. Global Kruskal–Wallis *p*-values are displayed within each panel, and pairwise Wilcoxon comparisons between categories are indicated above the boxes (ns, not significant; *p < 0.05; **p < 0.01; ***p < 0.001; ****p < 0.0001). Empty categories correspond to pathogen–antimicrobial combinations for which no isolates were classified in that susceptibility class. Across most combinations, MIC values increased significantly from susceptible to resistant categories, confirming the concordance between categorical antibiogram classification and quantitative MIC measurement.

Taken together, the MIC analysis indicates that antimicrobial resistance in the culturable tilapia pathogens was structured principally by pathogen identity and antibiotic, with significant pathogen-specific interactions and a significant additional contribution of developmental stage, in which the fry stage exhibited the highest MIC values. The concordance between categorical antibiogram classification and quantitative MIC values supports the internal consistency of the susceptibility data, while the pathogen × antibiotic interactions underscore the need for pathogen-specific, susceptibility-confirmed interpretation of antimicrobial data rather than empirical, cross-pathogen generalization in Nile tilapia aquaculture.

## 4 Discussion

This study presents the most comprehensive multi-pathogen epidemiological surveillance dataset reported to date for Nile tilapia aquaculture in Brazil, integrating detection positivity, spatiotemporal structure, temporal dynamics, co-detection, and antimicrobial resistance across 26,057 samples from three producing macro-regions over three years. The results reframe the traditional, *Streptococcus*-centered view of Brazilian tilapia health by revealing ISKNV and *Lactococcus* spp. as co-dominant agents, and by showing that pathogen circulation is structured principally along spatial and pathogen-specific temporal axes rather than by host developmental stage.

### 4.1 ISKNV and *Lactococcus*: the new paradigm of Brazilian tilapia health

The prominence of ISKNV (cumulative DPR 23.3%) and *Lactococcus* spp. (18.4%) among the seven agents surveyed represents a substantive change relative to the historically *Streptococcus*-dominated framework of Brazilian tilapia pathology (4, 41). ISKNV, first reported in Brazilian Nile tilapia in 2020 (10), has since established a stable, endemically compatible presence in the CW and SE macro-regions while apparently expanding into the NE, a trajectory consistent with dissemination through fingerling restocking, water-borne transmission, or wild-fish reservoirs (42, 43).

The stationarity of the ISKNV time series (ADF *p* ≤ 0.01), together with its sustained mean positivity, is consistent with a transition from an emerging to an established, endemically circulating agent, a pattern that would warrant permanent vigilance rather than emergency containment; because stationarity is a statistical property of the series and not a direct demonstration of endemic establishment, this interpretation is offered as the most parsimonious reading of the combined temporal and spatial evidence rather than as formal proof. The strong regional structuring of ISKNV detection is mechanistically coherent: geographic heterogeneity plausibly reflects differential infection history and biosecurity, and the documented higher susceptibility of younger cohorts to megalocytiviruses (44) is consistent with the elevated positivity observed in early production stages, even though developmental stage did not reach significance as an independent predictor within every individual pathogen.

Although *L. garvieae* was detected in Brazilian tilapia as early as 2009 (45), the present data indicate that *Lactococcus* spp., driven largely by *L. petauri* (6), is now among the most widely detected bacterial agents in the sector, reaching cumulative positivity (18.4%) comparable to ISKNV. Its pronounced regional structuring, with historically high CW positivity now declining while the SE sustains a high burden, is consistent with a pathogen that has already achieved endemic levels in affected basins within the study window. Recent multilocus sequence typing of Brazilian *Lactococcus* isolates revealed extensive genetic diversity, with *L. petauri* lineages unrelated to known tilapia and trout outbreak strains elsewhere (46), underscoring the value of continued molecular surveillance.

### 4.2 Streptococcus: persistent burden and geographic expansion

*Streptococcus* spp. (cumulative DPR 15.2%) remains a central epidemiological concern despite ranking third in overall positivity. For *Streptococcus* spp., non-stationarity reflected sustained directional drift against an expanding testing base. This trajectory, driven by a steady year-on-year increase and a marked emergence in the NE (0.0% in 2023 to 15.5% in 2025), is most parsimoniously explained by genuine geographic expansion rather than by intensified detection, although the non-probabilistic design means this remains an inference rather than a demonstrated cause. At the national level, *Streptococcus* DPR was shaped mainly by developmental stage in the pathogen-specific model, suggesting that once *Streptococcus* spp. is established in a production environment, the probability of clinical disease is determined by host and management-related factors rather than by broad temporal or spatial gradients alone (47–49).

This trajectory is consistent with Reis et al. (13), who detected *S. agalactiae* in the majority of surveyed production units, with serotype Ib distributed nationwide and serotype III more restricted to and increasing in the NE. Such serotype dynamics are further supported by genetic-diversity studies documenting substantial variation in virulence and resistance determinants among Brazilian isolates (5, 41, 47). Importantly, serotype Ia was not detected in any of the samples analyzed over the three-year period. The circulating *Streptococcus* population characterized here was dominated by serotype Ib, with serotype III and *S. iniae* as minor components.

This is epidemiologically significant because SaIa ST7 has recently emerged as an international threat to Latin American tilapia aquaculture, having been documented in acute streptococcosis outbreaks across six major tilapia-producing countries in the region between 2021 and 2025, where it forms a highly homogeneous cluster genetically related to reference strains previously isolated from tilapia farms in Asia, and where it has substantially undermined the competitiveness of the affected sectors through high mortality and production losses (21). The apparent absence of this clone from the Brazilian surveillance network, one of the largest tilapia producers in the region, represents a favorable but potentially fragile status. These findings underscore the need to maintain strict, serotype- and lineage-resolved surveillance for SaIa ST7 CC1 and to reinforce national biosecurity measures, particularly control over the cross-border movement of live fish and fingerlings, to prevent its introduction, establishment, and eventual dissemination within the Brazilian tilapia industry.

### 4.3 *Edwardsiella, Francisella, Flavobacterium, and Aeromonas*: restricted and stage-linked patterns

*Edwardsiella* spp. (cumulative DPR 10.2%) exhibited a consistent upward trend in the SE across the surveillance period, marking it as a priority emerging bacterial agent from a trend-surveillance perspective. The recent characterization of *E. anguillarum* as a distinct, highly virulent species in Brazilian tilapia, together with provisional epidemiological cut-off values for resistance interpretation, represents a critical diagnostic advance (9).

*F. orientalis* remained almost entirely confined to the SE macro-region, with stable positivity across years (7.9%, 11.6%, 12.6%), no sustained NE detection (0/187), and a stationary series (ADF *p* = 0.0169). Its high coefficient of variation (CV = 2.21) reflects sporadic detection around a low mean rather than directional change, exemplified by the isolated CW peak in 2024 (30.9%; 58/188) that reverted to zero. This endemic, equilibrium-phase pattern contrasts with Reis et al. (13), who reported higher detection in the S and SE during colder months, plausibly reflecting our limited S coverage and unstratified seasonal sampling (16).

*Flavobacterium* spp., now recognized as a species complex following recent taxonomic revision (8), yielded the highest cumulative positivity of all agents (21.6%) but on the smallest sampling base (1,014 tests), a dissociation that demands cautious interpretation. Its pronounced CW peak in 2024 followed by an abrupt decline, together with the wide interquartile ranges spanning much of the 0–1 scale, is most consistent with localized, management-driven events on few production units rather than a systemic epidemic. For *Flavobacterium* spp., non-stationarity arose from abrupt shifts generated by very small period-specific denominators and should be regarded as an artefact of sampling instability rather than evidence of directional epidemiological change.

*Aeromonas* spp. showed the lowest cumulative DPR (4.4%), consistent with its ecology as a ubiquitous opportunist whose detection tracks husbandry and environmental stressors rather than a specific co-infecting partner (7). Nonetheless, the 2022 emergence of a highly virulent, multidrug-resistant *A. veronii* in São Paulo (3), and reports of virulent *A. jandaei* causing mass mortality (50), demonstrate that sporadic high-impact events can punctuate this otherwise low-level background.

### 4.4 Co-detection structure: a weak ISKNV–streptococcal module

The co-detection analysis resolved the seven pathogens into four independent single-agent groups and a single multi-member module comprising ISKNV, *Streptococcus* spp., and *Lactococcus* spp. This module coincides exactly with the three most widely detected and geographically expanding agents and is consistent with field reports of ISKNV co-detected with bacterial pathogens in Brazilian tilapia (3, 6, 10). Crucially, however, all pairwise correlations were weak (*r* ≤ 0.12), so this association should be read as a consistent but low-magnitude epidemiological signal rather than deterministic co-occurrence (51).

Two non-exclusive mechanisms could generate this module. Megalocytiviruses induce systemic depletion of hematopoietic and lymphoid tissue (44), providing a plausible route by which a primary viral infection could lower the threshold for secondary bacterial disease (51). Alternatively, and indistinguishable from the first using presence/absence data, the three agents share thermal and stress-related drivers (high temperature, crowding, low dissolved oxygen) that can produce co-occurrence without direct biological interaction (6, 52). The weak correlation magnitudes and the cross-sectional, binary design mean that the immunosuppression hypothesis, while biologically attractive, remains inferential. Longitudinal sampling capable of ordering viral and bacterial detection in time, together with challenge studies, would be required to move from association to facilitation. Prospectively, should the association be confirmed, ISKNV surveillance could serve as an early indicator of elevated risk for streptococcal and lactococcal outbreaks under warm-season conditions.

### 4.5 Antimicrobial resistance and stewardship

The MIC modelling revealed that antimicrobial resistance was structured principally by pathogen identity and antibiotic, with pathogen-specific interactions not captured by categorical antibiogram classification alone. *Aeromonas* spp. emerged as the pathogen with the highest baseline MIC, whereas *Edwardsiella*, *Streptococcus*, and *Lactococcus* spp. showed comparatively lower MIC values relative to that reference. The concordance between resistant antibiogram classification and elevated MIC (estimate = +2.25; *p* < 0.001) confirms the internal consistency of the susceptibility data.

The significant pathogen × antibiotic interactions carry the most direct stewardship implications. *Edwardsiella* spp. and *Lactococcus* spp. showed markedly elevated MICs in combination with enrofloxacin and florfenicol, indicating that susceptibility to individual antibiotics cannot be generalized across pathogens and that empirical therapy is inappropriate without prior susceptibility confirmation. These findings are especially relevant given that only florfenicol and oxytetracycline are authorized for tilapia in Brazil (11), yet enrofloxacin and amoxicillin residues detected in commercial fillets indicate off-label use that may accelerate resistance (12).

In contrast to its influence on pathogen occurrence, where the effect was weak and detectable only at the assemblage level, developmental stage did exert a significant effect on MIC, with the fry stage associated with higher values. This dissociation, a negligible stage effect on detection but a significant one on resistance, indicates that host developmental stage acts on antimicrobial susceptibility rather than on pathogen occurrence, and merits confirmation in targeted studies.

Framed within a One Health perspective, these results reinforce that antimicrobial use in Brazilian tilapia aquaculture must be guided by pathogen identity and confirmed susceptibility rather than empirical practice, given the documented potential for resistant bacteria and determinants to disseminate along the food chain and into the environment (53, 54).

### 4.6 Implications for surveillance design

The convergence of the time-series, stationarity, and modelling analyses provides a descriptive basis to inform a practical national surveillance framework. Among the four aggregation frequencies evaluated, quarterly sampling yielded the lowest prediction error for every pathogen while preserving detection of the main temporal features, supporting it as a reasonable and cost-effective candidate interval rather than a formally optimized one; establishing a truly optimal interval would require prospective evaluation against defined detection targets. The stationary dynamics of five of the seven agents are compatible with the definition of provisional statistical control limits to help distinguish epidemic from endemic fluctuation, particularly for ISKNV and *Lactococcus* spp., once validated on independent data.

The non-stationary, apparently expanding behavior of *Streptococcus* spp., together with the late emergence signal shared by ISKNV and *Lactococcus* spp. in the NE, identifies that macro-region as a candidate priority for intensified monitoring, while recognizing that changing sampling effort could contribute to the observed trend. Because disease dynamics appeared to track biological macro-regions rather than administrative boundaries, surveillance could be usefully structured around production-system clusters, in line with risk-based approaches already being adopted in Brazil (48, 55), and with the harmonized frameworks deployed in salmon aquaculture in Norway and Chile (56–58).

Taken together, these findings position this dataset as a descriptive baseline that can inform the development of a structured and harmonized national health surveillance system for tilapia. To date, tilapia disease surveillance and sampling plans throughout Brazil and neighboring countries vary widely in terms of design and sample size, with a marked trend toward a risk-based approach. At the same time, several limitations qualify the findings. The DPR values derive from a structured but non-probabilistic, voluntary surveillance network, were not corrected for diagnostic sensitivity and specificity, use different observational units across modalities (pools vs. individual fish), and are weighted toward the SE, which contributed most tests. Inter-regional and inter-annual comparisons should therefore be read as relative trends rather than absolute population-level prevalence.

The stationarity classification summarizes series behavior but does not by itself prove endemic establishment, and the co-detection inferences rest on cross-sectional, binary data and weak correlations. The stage-specific MIC and detection estimates were constrained by uneven sample sizes, and coverage of the NE and CW macro-regions was sparse relative to the SE. A further limitation of the MIC analysis is that the model was fitted to the representative (median) MIC of each pathogen × antibiotic × antibiogram × stage combination rather than to individual isolates. This aggregation stabilizes estimation given the limited number of isolates per combination but discards within-combination isolate variability and does not model farm-level clustering of isolates, so the reported MIC effects should be read as combination-level associations rather than isolate-level effect sizes. These constraints define clear priorities for longitudinal, probabilistically designed follow-up studies.

Looking ahead, the trajectory of Brazilian tilapia health will depend on translating descriptive surveillance into anticipatory, decision-oriented monitoring. Three priorities emerge from the present data. First, detection capacity should be strengthened by harmonizing case definitions and diagnostic modalities across laboratories, expanding molecular panels to resolve species and serotypes (including routine screening for the *S. agalactiae* serotype Ia ST7 CC1 lineage), and integrating point-of-care and sequencing-based confirmation for emerging agents such as *E. anguillarum* and reclassified *Flavobacterium* species. Second, the marked under-representation of the NE and CW macro-regions relative to the SE, and of balanced production-stage sampling, should be corrected through a probabilistically designed, risk-based sampling frame that enables true prevalence estimation and seasonal stratification. Third, surveillance outputs should feed regionalized early-warning dashboards with provisional alert thresholds, coupled to pre-treatment susceptibility testing and cross-border biosecurity controls on live-fish and fingerling movements. Realizing these steps will require coordinated action among producers, diagnostic laboratories, and the competent authorities, and would move the sector from reactive outbreak response toward a predictive, One Health–aligned surveillance system for one of the world’s fastest-growing aquaculture industries.

## 5 Conclusion

This study presents the most comprehensive pathogen-level epidemiological surveillance dataset reported to date for Nile tilapia aquaculture in Brazil, integrating detection positivity, spatiotemporal structure, temporal dynamics, co-detection, and antimicrobial resistance across 26,057 samples from three macro-regions over three years (2023–2025). The results reframe the historically *Streptococcus*-centered view of Brazilian tilapia health, establishing ISKNV (cumulative DPR 23.3%) and *Lactococcus* spp. (18.4%) as co-dominant surveillance targets alongside *Streptococcus* spp. (15.2%).

Pathogen detection was structured principally along spatial and pathogen-specific temporal axes, with host developmental stage exerting a heterogeneous effect that was significant for some agents individually and weak but consistent at the assemblage level. Five of the seven agents displayed stationary, endemically compatible dynamics suitable for defining provisional baseline control thresholds, including *F. orientalis*, which remained anchored to the established Southeast production core. Two series were non-stationary, but for distinct reasons: *Streptococcus* spp. showed sustained directional drift against an expanding testing base, consistent with active geographic expansion into the Northeast, whereas non-stationarity in *Flavobacterium* spp. arose from abrupt shifts generated by very small period-specific denominators and is interpreted as sampling instability rather than directional epidemiological change. This distinction between genuine trend and denominator-driven variability is central to the operational use of these baselines. Among the frequencies evaluated, quarterly aggregation minimized forecast error without loss of detection capacity.

Co-detection was predominantly independent, with only a weak module linking the three dominant agents, likely reflecting shared environmental exposure. Antimicrobial resistance was structured by pathogen and antibiotic rather than geography, with significant pathogen × antibiotic interactions precluding empirical therapy without prior susceptibility confirmation; developmental stage influenced susceptibility (higher fry MICs) but not pathogen occurrence.

Notably, this large-scale effort found no evidence, by serotype-specific qPCR, of the recently described *S. agalactiae* serotype Ia (the ST7 CC1 clonal lineage described elsewhere) in the Brazilian units monitored, in contrast to its damaging emergence across six other Latin American producers, reinforcing the need for serotype- and lineage-resolved surveillance and strengthened biosecurity over cross-border fish movements. Collectively, and within the constraints of a structured but non-probabilistic design, these findings provide a descriptive, data-driven baseline to inform a national surveillance system organized around biological macro-regions, employing quarterly, stage-informed sampling, provisional region-specific alert thresholds, priority monitoring of the NE, and pre-treatment susceptibility testing, while establishing internationally comparable epidemiological baselines for one of the world’s fastest-growing aquaculture sectors. Key remaining challenges are to move from apparent to true prevalence through probabilistic sampling, to correct the current geographic and stage imbalances, to resolve pathogens to the species, serotype, and lineage level, and to validate the proposed alert thresholds and surveillance interval prospectively before they are adopted as national standards.

## Data Availability

All data generated or analyzed during this study are included in this article and its supplementary information files. The dataset that supports the findings of this study are available upon reasonable request from the corresponding author.

## References

[1] Arthur R. (ed). Review of the state of the world fishery resources - Inland fisheries. Fourth edition. FAO Fisheries and Aquaculture Circular, No. 942 Rev. 4. Rome, FAO and Abidjan, AfDB. (2024). doi: 10.4060/cd2169en

[2] Peixe BR. In: Anúario PABD, editor. Anuário Brasileiro da Piscicultura PEIXE BR 2025. São Paulo, SP - Brasil: PeixeBR - Associação Brasileira da Piscicultura (2025). p. 67.

[3] dos Santos SB Alarcon MF Ballaben AS Harakava R Galetti R Guimarães MC . First report of Aeromonas veronii as an emerging bacterial pathogen of farmed Nile tilapia (Oreochromis niloticus) in Brazil. Pathogens. (2023) 12:1020. doi: 10.3390/pathogens12081020, 37623980 PMC10459805

[4] Mian G Mian GF Godoy DT Leal CAG Yuhara TY Costa GM . Aspects of the natural history and virulence of *S. agalactiae* infection in Nile tilapia. Vet Microbiol. (2009) 136:180–3. doi: 10.1016/j.vetmic.2008.10.016 19042097

[5] Chideroli RT Amoroso N Mainardi RM Suphoronski SA de Padua SB Alfieri AF . Emergence of a new multidrug-resistant and highly virulent serotype of *Streptococcus agalactiae* in fish farms from Brazil. Aquaculture. (2017) 479:45–51. doi: 10.1016/j.aquaculture.2017.05.013

[6] Egger R.C. Rosa Júlio César Câmara Resende Luís Fernando Lara Pádua Santiago Benites de Oliveira Barbosa Fernanda Zerbini Mariana Taíse . Emerging fish pathogens *Lactococcus petauri* and *L. garvieae* in Nile tilapia (*Oreochromis niloticus*) farmed in Brazil Aquaculture (2023) 565:739093 doi: 10.1016/j.aquaculture.2022.739093

[7] Reis CS dos Santos JS Evangelista-Barreto NS. *Aeromonas* spp. in farmed fish in Brazil: a systematic literature review. Revista Multidisciplinar do Nordeste Mineiro. (2025) 21:1–20. doi: 10.61164/9r6ham53

[8] Janampa-Sarmiento PC Costa HL Rosa JCC Tavares GC Figueiredo HCP. Identification and phylogenetic analysis of Flavobacterium spp. associated with aquaculture fish diseased from Brazil. Pathogens. (2025) 14:219. doi: 10.3390/pathogens14030219, 40137703 PMC11944601

[9] Ferrari NA da Silva VCS Turini PG de Souza JF Mainardi RM da Silva MB . Phenotypic antimicrobial resistance profiles and provisional epidemiological cut-off values of Edwardsiella anguillarum isolated from farmed Nile Tilapia (Oreochromis niloticus) in Brazil, with exploratory data on Edwardsiella tarda. Microorganisms. (2026) 14:523. doi: 10.3390/microorganisms14030523, 41900283 PMC13029526

[10] Figueiredo HCP Tavares GC Dorella FA Rosa JCC Marcelino SAC Pierezan F . First report of infectious spleen and kidney necrosis virus in Nile tilapia in Brazil. Transbound Emerg Dis. (2022) 69:3008–15. doi: 10.1111/tbed.14217, 34223695

[11] Assane IM Gozi KS Valladão GMR Pilarski F. Combination of antimicrobials as an approach to reduce their application in aquaculture: emphasis on the use of thiamphenicol/florfenicol against *Aeromonas hydrophila*. Aquaculture. (2019) 507:238–45. doi: 10.1016/j.aquaculture.2019.04.021

[12] Bortolotte AR Daniel D Reyes FGR. Occurrence of antimicrobial residues in tilapia (Oreochromis niloticus) fillets produced in Brazil and available at the retail market. Food Res Int. (2021) 140:109865. doi: 10.1016/j.foodres.2020.109865, 33648183

[13] Reis FYT Rosa JCC Tavares GC Moreira GA Nicolino RR Figueiredo HCP. Seasonal and regional dynamics of *Streptococcus agalactiae* and *Francisella orientalis* infections in Brazilian tilapia: a six-year national study. Transbound Emerg Dis. (2026) 2026:5995172. doi: 10.1155/tbed/5995172

[14] Bennett RH Ellender BR Mäkinen T Miya T Pattrick P Wasserman RJ . Ethical considerations for field research on fishes. Koedoe. (2016) 58:1–15. doi: 10.4102/koedoe.v58i1.1353, 42762356

[15] Jenkins JA Bart HL Bowker JD Bowser P MacMillan JR Nickum JG . Guidelines for use of fishes in research: revised and expanded: Fisheries, v. 39, no. 9, (2014). p. 415–416.

[16] Soto E Hawke JP Fernandez D A Morales J. Francisella sp., an emerging pathogen of tilapia, Oreochromis niloticus (L.), in Costa Rica. J Fish Dis. (2009) 32:713–22. doi: 10.1111/j.1365-2761.2009.01070.x, 19515205

[17] Piamsomboon P Jaresitthikunchai J Hung TQ Roytrakul S Wongtavatchai J. Identification of bacterial pathogens in cultured fish with a custom peptide database constructed by matrix-assisted laser desorption/ionization time-of-flight mass spectrometry (MALDI-TOF MS). BMC Vet Res. (2020) 16:52. doi: 10.1186/s12917-020-2274-1, 32046727 PMC7014616

[18] Assis GBN Pereira FL Zegarra AU Tavares GC Leal CA Figueiredo HCP. Use of MALDI-TOF mass spectrometry for the fast identification of gram-positive fish pathogens. Front Microbiol. (2017) 8:1492. doi: 10.3389/fmicb.2017.01492 PMC5552964 28848512

[19] Rimmer AE Becker JA Tweedie A Whittington RJ. Development of a quantitative polymerase chain reaction (qPCR) assay for the detection of dwarf gourami iridovirus (DGIV) and other megalocytiviruses and comparison with the office international des epizooties (OIE) reference PCR protocol. Aquaculture. (2012) 358:155–63. doi: 10.1016/j.aquaculture.2012.06.034

[20] Phasunon R Taengphu S Panphut W Chatchaiphan S Dong HT Senapin S. Improving the diagnosis of Streptococcus iniae using a novel probe-based qPCR assay combined with an enrichment step. J Fish Dis. (2023) 46:1391–401. doi: 10.1111/jfd.13857, 37723600

[21] Rozas-Serri M Fernandez-Alarcon M Miyoko-Natori M Galetti R Harakava R Cardoso-Guimarães M . Streptococcus agalactiae serotype Ia ST7 CC1 in farmed Nile Tilapia in Latin America: age-dependent disease expression and antimicrobial susceptibility of an emerging clonal lineage. Pathogens. (2026) 15:545. doi: 10.3390/pathogens15050545, 42198670 PMC13209410

[22] Su YL Feng J Li YW Bai JS Li AX. Development of a quantitative PCR assay for monitoring Streptococcus agalactiae colonization and tissue tropism in experimentally infected tilapia. J Fish Dis. (2016) 39:229–38. doi: 10.1111/jfd.12358, 25858765

[23] Shahin K Mukkatira K Yazdi Z Richey C Kwak K Heckman TI . Development of a quantitative polymerase chain reaction assay for detection of the aetiological agents of piscine lactococcosis. J Fish Dis. (2022) 45:847–59. doi: 10.1111/jfd.13610, 35306674

[24] Griffin MJ Goodwin AE Merry GE Liles MR Williams MA Ware C . Rapid quantitative detection of Aeromonas hydrophila strains associated with disease outbreaks in catfish aquaculture. J Vet Diagn Invest. (2013) 25:473–81. doi: 10.1177/1040638713494210, 23847227

[25] Soto E Bowles K Fernandez D Hawke JP. Development of a real-time PCR assay for identification and quantification of the fish pathogen Francisella noatunensis subsp. orientalis. Dis Aquat Org. (2010) 89:199–207. doi: 10.3354/dao02204, 20481087

[26] Reichley SR Ware C Greenway TE Wise DJ Griffin MJ. Real-time polymerase chain reaction assays for the detection and quantification of Edwardsiella tarda, Edwardsiella piscicida, and Edwardsiella piscicida–like species in catfish tissues and pond water. J Vet Diagn Invest. (2015) 27:130–9. doi: 10.1177/1040638714566672, 25613040

[27] Gibbs GD Griffin MJ Mauel MJ Lawrence ML. Validation of a quantitative PCR assay for the detection of 2 Flavobacterium columnare genomovars. J Vet Diagn Invest. (2020) 32:356–62. doi: 10.1177/1040638720915760, 32310022 PMC7377616

[28] Yang CG Wang XL Tian J Liu W Wu F Jiang M . Evaluation of reference genes for quantitative real-time RT-PCR analysis of gene expression in Nile tilapia (Oreochromis niloticus). Gene. (2013) 527:183–92. doi: 10.1016/j.gene.2013.06.013, 23792389

[29] Caraguel CG Caraguel CGB Stryhn H Gagné N Dohoo IR Hammell KL. Selection of a cutoff value for real-time polymerase chain reaction results to fit a diagnostic purpose: analytical and epidemiologic approaches. J Vet Diagn Invest. (2011) 23:2–15. doi: 10.1177/104063871102300102, 21217022

[30] CLSI, C.a.L.S.I. Performance Standards for Antimicrobial Susceptibility Testing. 35th ed. CLSI supplement M100. Wayne, PA, USA: Clinical and Laboratory Standards Institute (2025).

[31] CLSI, C.a.L.S.I. Performance Standards for Antimicrobial Disk and Dilution Susceptibility Tests for Bacteria Isolated from Animals. 6th ed. CLSI Standard VET01. Wayne, PA, USA: Clinical and Laboratory Standards Institute (2024).

[32] CLSI, C.a.L.S.I. Methods for Dilution Antimicrobial Susceptibility Tests for Bacteria that Grow Aerobically. Wayne, PA: Clinical and Laboratory Standards Institute (2024).

[33] Hyndman RJ Khandakar Y. Automatic time series forecasting: the forecast package for R. J Stat Softw. (2008) 27:1–22. doi: 10.18637/jss.v027.i03

[34] Trapletti A. Hornik K. Tseries: Time series analysis and computational finance. R package version 0.10-60. (2026). Available online at: https://cran.r-project.org/package=tseries

[35] Wei T. Simko V. R package ‘Corrplot’: Visualization of a Correlation Matrix (Version 0.95). (2024). Available online at: https://github.com/taiyun/corrplot

[36] Csardi G Nepusz T. The igraph software. Complex Syst. (2006) 1695:1–9.

[37] Blondel VD Guillaume J-L Lambiotte R Lefebvre E. Fast unfolding of communities in large networks. J Stat Mech-Theory Exp. (2008) 2008:P10008. doi: 10.1088/1742-5468/2008/10/P10008

[38] Fruchterman TM Reingold EM. Graph drawing by force-directed placement. Softw Pract Exper. (1991) 21:1129–64. doi: 10.1002/spe.4380211102

[39] Team, R.C. R: A Language and Environment for Statistical Computing. Vienna, Austria: R foundation for statistical computing (2021).

[40] Robinson D Hayes A Couch S Hvitfeldt E *broom: Convert Statistical Objects into Tidy Tibbles.* R package version 1.0.13. (2026). Available online at: https://broom.tidymodels.org/

[41] Barony GM Tavares GC Pereira FL Carvalho AF Dorella FA Leal CAG . Large-scale genomic analyses reveal the population structure and evolutionary trends of Streptococcus agalactiae strains in Brazilian fish farms. Sci Rep. (2017) 7:13538. doi: 10.1038/s41598-017-13228-z, 29051505 PMC5648781

[42] de Oliveira Viadanna PH Brunner J. Evaluating responses to epidemics of infectious spleen and kidney necrosis virus in farmed tilapia (*Oreochromis niloticus*). Ecol Model. (2025) 510:111270. doi: 10.1016/j.ecolmodel.2025.111270

[43] Ramírez-Paredes JG Paley RK Hunt W Feist SW Stone DM Field TR . First detection of infectious spleen and kidney necrosis virus (ISKNV) associated with massive mortalities in farmed tilapia in Africa. Transbound Emerg Dis. (2021) 68:1550–63. doi: 10.1111/tbed.13825, 32920975 PMC8246855

[44] Subramaniam K Gotesman M Smith CE Steckler NK Kelley KL Groff JM . Megalocytivirus infection in cultured Nile tilapia Oreochromis niloticus. Dis Aquat Org. (2016) 119:253–8. doi: 10.3354/dao02985, 27225209

[45] Evans J Klesius P Shoemaker C. First isolation and characterization of Lactococcus garvieae from Brazilian Nile tilapia, Oreochromis niloticus (L.), and pintado, Pseudoplathystoma corruscans (Spix & Agassiz). J Fish Dis. (2009) 32:943–51. doi: 10.1111/j.1365-2761.2009.01075.x, 19531061

[46] Tavares G.C. Carneiro Sarah Portes Barbanti Angelo Carlo Chaparro Rosário Angélica Emanuely Costa do Matos Helena Caldeira Maia Cynthia Rafaela Monteiro da Silva . Multilocus sequence typing reveals new insights into the population structure and genetic diversity of *Lactococcus* spp. from Brazilian fish Microorganisms (2026) 14.:1131 doi: 10.3390/microorganisms14051131 PMC13209592 42197516

[47] Assane IM de Oliveira Neto RR de Abreu Reis Ferreira D do Vale Oliveira A Hashimoto DT Pilarski F. Genetic diversity, virulence genes, antimicrobial resistance genes, and antimicrobial susceptibility of group B Streptococcus (GBS) associated with mass mortalities of cultured Nile tilapia in Brazil. Microb Pathog. (2025) 205:107664. doi: 10.1016/j.micpath.2025.107664, 40324602

[48] Delphino MK Delphino MKVC Leal CAG Gardner IA Assis GBN Roriz GD . Seasonal dynamics of bacterial pathogens of Nile tilapia farmed in a Brazilian reservoir. Aquaculture. (2019) 498:100–8. doi: 10.1016/j.aquaculture.2018.08.023

[49] Oliveira-Neto RR Mastrochirico-Filho VA Assane IM Ariede RB Freitas MV Agudelo JFG . Resistance of juvenile Nile tilapia *Oreochromis niloticus* from Brazilian populations to *Streptococcus agalactiae* (serotype Ib and ST-NT). Front Aquac. (2024) 3:1354029. doi: 10.3389/faquc.2024.1354029

[50] Assane IM de Sousa EL Valladão GMR Tamashiro GD Criscoulo-Urbinati E Hashimoto DT . Phenotypic and genotypic characterization of *Aeromonas jandaei* involved in mass mortalities of cultured Nile tilapia, *Oreochromis niloticus* (L.) in Brazil. Aquaculture. (2021) 541:736848. doi: 10.1016/j.aquaculture.2021.736848

[51] Kotob MH Menanteau-Ledouble S Kumar G Abdelzaher M el-Matbouli M. The impact of co-infections on fish: a review. Vet Res. (2016) 47:98. doi: 10.1186/s13567-016-0383-4, 27716438 PMC5050641

[52] Kayansamruaj P Pirarat N Hirono I Rodkhum C. Increasing of temperature induces pathogenicity of *Streptococcus agalactiae* and the up-regulation of inflammatory related genes in infected Nile tilapia (*Oreochromis niloticus*). Vet Microbiol. (2014) 172:265–71. doi: 10.1016/j.vetmic.2014.04.013 24856132

[53] Haenen OL Haenen OLM Dong HT Hoai TD Crumlish M Karunasagar I . Bacterial diseases of tilapia, their zoonotic potential and risk of antimicrobial resistance. Rev Aquac. (2023) 15:154–85. doi: 10.1111/raq.12743

[54] Schar D Zhao C Wang Y Larsson DGJ Gilbert M van Boeckel TP. Twenty-year trends in antimicrobial resistance from aquaculture and fisheries in Asia. Nat Commun. (2021) 12:5384. doi: 10.1038/s41467-021-25655-8, 34508079 PMC8433129

[55] Raposo RS Delphino MKVC Rezende LPO Sant’Ana FJF. Tilapia disease surveillance and sampling plans for epidemiological surveys and monitoring: a review. Pesqui Vet Bras. (2025) 45:e07593. doi: 10.1590/1678-5150-pvb-7593

[56] Bondad-Reantaso MG Fejzic N MacKinnon B Huchzermeyer D Seric‐Haracic S Mardones FO . A 12-point checklist for surveillance of diseases of aquatic organisms: a novel approach to assist multidisciplinary teams in developing countries. Rev Aquac. (2021) 13:1469–87. doi: 10.1111/raq.12530

[57] Delphino MK Delphino MKVC Mardones FO Neumann Heise J Gallardo A Jimenez D . Cost-effectiveness of longitudinal surveillance for *Piscirickettsia salmonis* using qPCR in Atlantic salmon farms (*Salmo salar*) in Chile. J Fish Dis. (2021) 44:315–26. doi: 10.1111/jfd.13285, 33180978

[58] Oliveira VH Oliveira VHS Dórea FC Dean KR Bang Jensen B. Exploring options for syndromic surveillance in aquaculture: outbreak detection of salmon pancreas disease using production data from Norwegian farms. Transbound Emerg Dis. (2024) 2024:9861677. doi: 10.1155/2024/9861677, 40303021 PMC12017065

