# Supplementary material for: Epidemiological surveillance reveals major pathogen dynamics in Brazilian Nile tilapia aquaculture from 2023 to 2025
Source: Front Vet Sci. 2026 Oct 19;13:1944605. doi: 10.3389/fvets.2026.1944605 (PMC13726547; doi:10.3389/fvets.2026.1944605)
Supplement: Supplementary file 1 [file Supplementary_file_1.DOCX]

**Supplementary Information**

**Table S1.** Composition of the diagnostic surveillance network by Brazilian state, with the legal administrative region and the study macro-region to which each state was assigned (North – Northeast; South – Southeast). Companies (empresas) are distinct client records; culture sites are distinct culture-site localities (the unit modelled as the Site random effect). Several companies may operate within a shared culture-site locality, so companies exceed sites in some states. Column totals are the sum across states.

| **State** | **Legal region** | **Study macro-region** | **Companies (empresas)** | **Culture sites** |
| --- | --- | --- | --- | --- |
| Ceará (CE) | Northeast | NE | 1 | 1 |
| Bahia (BA) | Northeast | NE | 9 | 6 |
| Pernambuco (PE) | Northeast | NE | 6 | 4 |
| Piauí (PI) | Northeast | NE | 1 | 1 |
| Alagoas (AL) | Northeast | NE | 2 | 3 |
| Maranhão (MA) | Northeast | NE | 1 | 1 |
| Distrito Federal (DF) | Central-West | CW | 4 | 2 |
| Goiás (GO) | Central-West | CW | 14 | 9 |
| Mato Grosso do Sul (MS) | Central-West | CW | 16 | 7 |
| Mato Grosso (MT) | Central-West | CW | 1 | 1 |
| São Paulo (SP) | Southeast | SE | 63 | 45 |
| Minas Gerais (MG) | Southeast | SE | 71 | 19 |
| Espírito Santo (ES) | Southeast | SE | 1 | 1 |
| Paraná (PR) | South | SE | 60 | 33 |
| Santa Catarina (SC) | South | SE | 25 | 14 |
| Tocantins (TO) | North | NE | 5 | 7 |
| **Total** |  |  | **280** | **154** |

**Table S2.** Analysis of deviance of nested fixed-effects generalized linear models (GLM; quasibinomial, logit link) for detection positivity rate (DPR) across all pathogens combined, compared by sequential analysis of deviance. Res. df, residual degrees of freedom; ∆df, change in degrees of freedom; ∆Deviance, change in deviance. The interpretable additive model (Model 3) is shown in bold.

| **Model** | **Structure** | **Res. df** | **Deviance** | **∆df** | **∆Deviance** | **F** | **Pr(>F)** | **Pr(>Chi)** |
| --- | --- | --- | --- | --- | --- | --- | --- | --- |
| Model 1 | DPR ~ Pathogeno/(Macro-Region + Year + Stage) | 16771 | 13709 |  |  |  |  |  |
| Model 2 | DPR ~ Pathogeno/Year + (Macro-Region + Stage) | 16795 | 55354 | -24 | -41645 | 348.1325 | < 2,2e-16 | < 2,2e-16 |
| **Model 3** | **DPR ~ Pathogeno + Year + Macro-Region + Stage** | 16807 | 55722 | -12 | -368 | 6.1479 | 6.649e-11 | 6.257e-11 |
| Model 4 | DPR ~ Year/(Pathogeno * Macro-Region) + Stage | 16757 | 54083 | 50 | 1639 | 6.577 | < 2,2e-16 | < 2,2e-16 |
| Model 5 | DPR ~ Year + (Pathogeno/Macro-Region) + Stage | 16795 | 55379 | -38 | -1296 | 6.8413 | < 2,2e-16 | < 2,2e-16 |

**Table S3.** Comparison of nested generalized linear mixed models (GLMM; binomial, logit link) by information criteria, for selection of the random-effect structure. npar, number of parameters; AIC/BIC, information criteria; logLik, log-likelihood. The best-supported model (Model 1; lowest AIC = 11,779) is shown in bold.

| **Model** | **Model structure** | **npar** | **AIC** | **BIC** | **logLik** | **deviance** | **Chisq** | **Df** | **Pr(>Chisq)** |
| --- | --- | --- | --- | --- | --- | --- | --- | --- | --- |
| **Model 1** | **c(positive, negative) ~ Pathogen + Macro Region + Year + Fase + (1 \| Epi Week) + (1 \| Site) + (1 \| ID)** | 16 | **11779** | 11902 | -5873 | 11747 | 581.42 | 0 |  |
| Model 2 | c(positive, negative) ~ Pathogen + Macro-Region + Year + Fase + (1 \| Epi Week) + (1 \| Pathogen:Macro-Region) + (1 \| Site) + (1 \| ID) | 17 | 11781 | 11912 | -5873 | 11747 | 0 | 1 | 1 |
| Model 3 | c(positive, negative) ~ Pathogen + Macro-Region + Year + Fase + (1 \| Epi Week) + (1 \| Pathogen:Macro-Region) + (1 \| Site) | 16 | 12360 | 12484 | -6164 | 12328 | 66158.83 | 1 | <2e-16 |
| Model 4 | c(positive, negative) ~ Pathogen + Macro-Region + Year + Fase + (1 \| Epi Week) + (1 \| Pathogen:Macro-Region) | 15 | 13745 | 13861 | -6857 | 13715 |  |  |  |
| Model 5 | c(positive, negative) ~ Year + Pathogen + Macro-Region + Fase + (1 \| Pathogen:Macro-Region) + (1 \| Site) | 15 | 78517 | 78633 | -39243 | 78487 | 0 | 0 |  |

**Table S4.** Selection of candidate time-series (ARIMA) models for the DPR of each pathogen, ranked by information criteria; the three best-supported models per pathogen are shown, with the best in bold. AIC, Akaike information criterion; BIC, Bayesian information criterion; logLik, log-likelihood; σ², residual variance.

| **Pathogen** | **Model** | **n obs** | **AIC** | **BIC** | **logLik** | **σ²** |
| --- | --- | --- | --- | --- | --- | --- |
| *Aeromonas* | **AutoRegresivo (AR) ARIMA(0,0,0)** | 61 | **-35.9** | -31.7 | 20 | 0.176 |
|  | AutoRegresivo (AR) ARIMA(1,0,0) | 61 | -34.5 | -28.2 | 20.2 | 0.177 |
|  | Media Simple (MS) ARIMA(0,0,1) | 61 | -34.3 | -28 | 20.2 | 0.177 |
| *Edwardsiella* | **AutoRegresivo (AR) ARIMA(0,0,0)** | 79 | **-109** | -104 | 56.4 | 0.119 |
|  | AutoRegresivo (AR) ARIMA(1,0,0) | 79 | -107 | -99.6 | 56.4 | 0.12 |
|  | Media Simple (MS) ARIMA(0,0,1) | 79 | -107 | -99.6 | 56.4 | 0.12 |
| *Flavobacterium* | **AutoRegresivo (AR) ARIMA(0,0,0)** | 41 | **9.5** | 13 | -2.8 | 0.262 |
|  | AutoRegresivo (AR) ARIMA(1,0,0) | 41 | 10.7 | 15.9 | -2.4 | 0.263 |
|  | Media Simple (MS) ARIMA(0,0,1) | 41 | 10.9 | 16 | -2.4 | 0.263 |
| *Francisella* | **AutoRegresivo (AR) ARIMA(0,0,0)** | 61 | **-18.9** | -14 | 11.5 | 0.213 |
|  | AutoRegresivo (AR) ARIMA(1,0,0) | 61 | -17.1 | -9.7 | 11.5 | 0.214 |
|  | Media Simple (MS) ARIMA(0,0,1) | 61 | -17.1 | -9.7 | 11.5 | 0.214 |
| *ISKNV* | **AutoRegresivo (AR) ARIMA(0,0,0)** | 131 | **56.4** | 65 | -25.2 | 0.295 |
|  | AutoRegresivo (AR) ARIMA(1,0,0) | 131 | 57.3 | 65.9 | -25.7 | 0.297 |
|  | Media Simple (MS) ARIMA(0,0,1) | 131 | 57.3 | 66 | -25.7 | 0.297 |
| *Lactococcus* | **AutoRegresivo (AR) ARIMA(0,0,0)** | 99 | **45.5** | 55.9 | -18.7 | 0.296 |
|  | AutoRegresivo (AR) ARIMA(1,0,0) | 99 | 49.3 | 57.1 | -21.6 | 0.304 |
|  | Media Simple (MS) ARIMA(0,0,1) | 99 | 49.3 | 57.1 | -21.6 | 0.304 |
| *Streptococcus* | **AutoRegresivo (AR) ARIMA(0,0,0)** | 101 | **-28.3** | -20.5 | 17.2 | 0.206 |
|  | AutoRegresivo (AR) ARIMA(1,0,0) | 101 | -28.3 | -20.5 | 17.2 | 0.206 |
|  | Media Simple (MS) ARIMA(0,0,1) | 101 | -26.7 | -18.9 | 16.4 | 0.208 |

**Table S5.** Predictive performance of the selected time-series models for each pathogen across four temporal aggregation frequencies. MSE, mean squared error; MAE, mean absolute error; Residual SD, residual standard deviation; Shapiro p, Shapiro–Wilk normality p-value; N, number of observation periods. The quarterly scale (lowest prediction error) is shown in bold.

| **Pathogen** | **Frequency** | **Model** | **MSE** | **MAE** | **Residual SD** | **Shapiro p** | **N** |
| --- | --- | --- | --- | --- | --- | --- | --- |
| *Aeromonas* | 4 | **Quarterly** | **0.0196** | 0.0919 | 0.141 | 4.4e-05 | 61 |
|  | 12 | Monthly | 0.0192 | 0.0962 | 0.1397 | 0.0018 | 61 |
|  | 13 | Four-Week | 0.0215 | 0.0987 | 0.1479 | 1.2e-05 | 61 |
|  | 52 | Weekly | 0.0304 | 0.1076 | 0.1759 | < 1e-10 | 61 |
| *Edwardsiella* | 4 | **Quarterly** | **0.0093** | 0.0678 | 0.0971 | 5.9e-06 | 79 |
|  | 12 | Monthly | 0.0114 | 0.0821 | 0.1074 | 8.6e-05 | 79 |
|  | 13 | Four-Week | 0.0108 | 0.0771 | 0.1046 | 1.9e-05 | 79 |
|  | 52 | Weekly | 0.0141 | 0.0896 | 0.1193 | < 1e-10 | 79 |
| *Flavobacterium* | 4 | **Quarterly** | **0.0311** | 0.1376 | 0.1786 | 0.1404 | 41 |
|  | 12 | Monthly | 0.0431 | 0.1593 | 0.2101 | 0.0922 | 41 |
|  | 13 | Four-Week | 0.0442 | 0.1678 | 0.2127 | 0.0436 | 41 |
|  | 52 | Weekly | 0.067 | 0.2045 | 0.2621 | 1.1e-06 | 41 |
| *Francisella* | 4 | **Quarterly** | **0.0274** | 0.0981 | 0.1666 | 2.8e-09 | 86 |
|  | 12 | Monthly | 0.0313 | 0.1124 | 0.178 | 1.0e-10 | 86 |
|  | 13 | Four-Week | 0.0319 | 0.1073 | 0.1797 | 2.0e-10 | 86 |
|  | 52 | Weekly | 0.0449 | 0.1273 | 0.213 | < 1e-10 | 86 |
| *ISKNV* | 4 | **Quarterly** | **0.0494** | 0.1738 | 0.223 | 8.5e-04 | 131 |
|  | 12 | Monthly | 0.0673 | 0.2011 | 0.2603 | 6.2e-05 | 131 |
|  | 13 | Four-Week | 0.0707 | 0.2094 | 0.267 | 1.6e-04 | 131 |
|  | 52 | Weekly | 0.0878 | 0.2354 | 0.2974 | 2.0e-09 | 131 |
| *Lactococcus* | 4 | **Quarterly** | **0.06** | 0.1809 | 0.2462 | 3.3e-05 | 99 |
|  | 12 | Monthly | 0.0705 | 0.1974 | 0.2669 | 3.6e-06 | 99 |
|  | 13 | Four-Week | 0.0708 | 0.2019 | 0.2674 | 1.1e-05 | 99 |
|  | 52 | Weekly | 0.0907 | 0.2205 | 0.3026 | < 1e-10 | 99 |
| *Streptococcus* | 4 | **Quarterly** | **0.0206** | 0.0984 | 0.1442 | 1.2e-04 | 101 |
|  | 12 | Monthly | 0.0296 | 0.1252 | 0.1729 | 2.0e-06 | 101 |
|  | 13 | Four-Week | 0.0306 | 0.1219 | 0.1759 | 3.5e-07 | 101 |
|  | 52 | Weekly | 0.0436 | 0.1452 | 0.2097 | 2.1e-08 | 101 |

**Table S6.** Sequential deviance analysis of minimum inhibitory concentration (MIC) values, evaluating developmental stage, pathogen identity, antibiotic, antibiogram category, and their interactions. Predictors were added sequentially; each row reports the change in deviance attributable to that term. Resid. Df, residual degrees of freedom; Resid. Dev., residual deviance. Significance codes: *** p < 0.001, ** p < 0.01, * p < 0.05, . p < 0.10.

| **Predictor** | **Df** | **Deviance** | **Resid. Df** | **Resid. Dev** | **Pr(>Chisq)** |
| --- | --- | --- | --- | --- | --- |
| NULL |  |  | 159 | 577.1 |  |
| **Stage** | 2 | 30.214 | 157 | 546.9 | 0.0116 * |
| **Pathogen** | 3 | 122.518 | 154 | 424.4 | 0.0014 ** |
| **Antibiotic** | 3 | 134.252 | 151 | 290.1 | 0.0002 *** |
| **Antibiogram** | 2 | 115.606 | 149 | 174.5 | < 0.001 *** |
| **Pathogen × Antibiotic** | 9 | 22.861 | 140 | 151.7 | 0.0606 . |
| **Pathogen × Antibiogram** | 6 | 17.816 | 134 | 133.86 | 0.0471 * |

**Table S7.** Comparison of candidate models for minimum inhibitory concentration (MIC) by information criteria (ten candidate structures), from which the selected model (Model 6) was retained. df, model degrees of freedom; AIC, Akaike information criterion; Res. Df, residual degrees of freedom; RSS, residual sum of squares; ∆df, change in degrees of freedom. The selected model is shown in bold.

| **Model** | **Structure** | **df** | **AIC** | **Res. Df** | **RSS** | **∆df** | **Sum of Sq** | **Pr(>Chi)** |
| --- | --- | --- | --- | --- | --- | --- | --- | --- |
| Model 1 | MIC ~ Pathogen + Macro-Region + Stage/(Antibiotic × Antibiogram) | 31 | 1464 | 130 | 59879.4 |  |  |  |
| Model 2 | MIC ~ Pathogen + Macro-Region + Stage + (Antibiotic × Antibiogram) | 21 | 1480.4 | 140 | 75131.5 | -10 | -15252.11 | 0 |
| Model 3 | MIC ~ Stage × Pathogen + Macro-Region + (Antibiotic × Antibiogram) | 24 | 1480.6 | 137 | 72471.1 | 3 | 2660.33 | 0 |
| Model 4 | MIC ~ Pathogen + Stage + (Antibiotic × Antibiogram) | 18 | 1477 | 143 | 76384.2 | -6 | -3913.1 | 0 |
| Model 5 | MIC ~ Pathogen + Stage/(Antibiotic × Antibiogram) | 28 | 1461.1 | 133 | 61050.1 | 10 | 15334.18 | 0 |
| **Model 6** | **MIC ~ Stage + Pathogen/(Antibiotic × Antibiogram)** | 36 | **785.8** | 125 | 124.9 | 8 | 60925.15 | 0 |
| Model 7 | MIC ~ Stage + Pathogen + Pathogen × Antibiotic + Pathogen × Antibiogram | 27 | 780.3 | 134 | 133.9 | -9 | -8.94 | 0.4426 |
| Model 8 | MIC ~ Stage + Pathogen + Antibiogram + Pathogen × Antibiotic + Pathogen × Antibiogram + Pathogen/Antibiotic/Antibiogram | 36 | 785.8 | 125 | 124.9 | 9 | 8.94 | 0.4426 |
| Model 9 | MIC ~ Pathogen/(Antibiotic × Antibiogram) | 34 | 792.9 | 127 | 132.9 | -2 | -7.95 | 0.0188 |
| Model 10 | MIC ~ Pathogen + Antibiotic + Antibiogram + Pathogen × Antibiotic + Pathogen × Antibiogram + Pathogen/(Antibiogram × Antibiotic) | 34 | 792.9 | 127 | 132.9 |  |  |  |

**Supplementary Figures**

**Figure S1.** Regional dispersion of the detection positivity rate (DPR) by pathogen and macro-region (raincloud plots: jittered points, box, and density). Complements the regional dynamics summarized in Figure 1 and Table 1.

**Figure S2.** Year-on-year dispersion of individual DPR values by pathogen (raincloud plots, 2023–2025), underlying the temporal trends shown in Figure 3.

**Figure S3.** Distribution of DPR by developmental stage (Adult, Juvenile, Fry), macro-region, and year for each pathogen (box-and-jitter plots). Illustrates the uneven stage representation referred to in the text.

**Figure S4.** Distribution of DPR by developmental stage (Adult, Juvenile, Fry) for each pathogen (raincloud plots).
